# Chemical Stability and Characterization of Degradation Products of Blends of 1-(2-Hydroxyethyl)pyrrolidine and 3-Amino-1-propanol

**DOI:** 10.1021/acs.iecr.2c03068

**Published:** 2022-12-19

**Authors:** Solrun Johanne Vevelstad, Andreas Grimstvedt, Maxime François, Hanna K. Knuutila, Geir Haugen, Merete Wiig, Kai Vernstad

**Affiliations:** † SINTEF Industry, 7465Trondheim, Norway; ‡ Department of Chemical Engineering, NTNU, NO-7491Trondheim, Norway

## Abstract

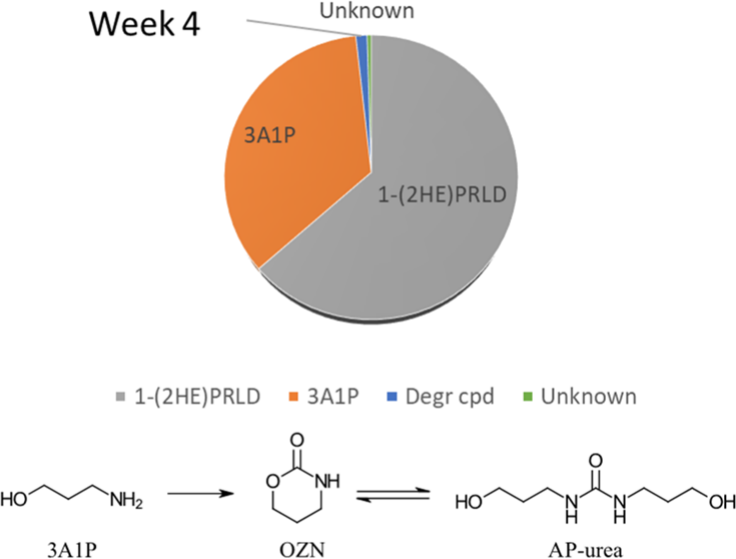

Aqueous amine solvents are used to capture CO_2_ from various flue gas sources. In this work, the chemical stability of a blend of 3-amino-1-propanol (3A1P) and 1-(2-hydroxyethyl)pyrrolidine [1-(2HE)PRLD] was studied. The chemical stability tests were conducted both in batch and cycled systems using various oxygen and NOx concentrations, additives (iron), and temperatures. In the thermal degradation experiments with CO_2_ present, the blend was more stable than the primary amines [(3A1P or monoethanolamine (MEA)] but less stable than the tertiary amine 1-(2HE)PRLD alone. Similar stability was observed between MEA, 3A1P, and the blend in the batch experiments at medium oxygen concentration (21% O_2_) and no iron present. 1-(2HE)PRLD was more stable. However, the presence of high oxygen concentration (96% O_2_) and iron reduced the stability of 1-(2HE)PRLD significantly. Furthermore, in the case of the blend, the chemical stability increased with increasing promoter concentration in batch experiments. During the cyclic experiment, the amine loss for the blend was similar to what was previously observed for MEA (30 wt %) under the same conditions. A thorough mapping of degradation compounds in the solvent and condensate samples resulted in the identification and quantification of 30 degradation compounds. The major components in batch and cycled experiments varied somewhat, as expected. In the cyclic experiments, the major components were ammonia, 3-(methylamino)-1-propanol (methyl-AP), *N*,*N*′-bis(3-hydroxypropyl)-urea (AP-urea), pyrrolidine, formic acid (formate), and *N*-(3-hydroxypropyl)-glycine (HPGly). Finally, in this paper, formation pathways for the eight degradation compounds (1,3-oxazinan-2-one, AP-urea, 3-[(3-aminopropyl)amino]-1-propanol, tetrahydro-1-(3-hydroxypropyl)-2(1*H*)-pyrimidinone, methyl-AP, *N*-(3-hydroxypropyl)-formamide, *N*-(3-hydroxypropyl)-β-alanine, and HPGly) are suggested.

## 1 Introduction

Carbon capture and storage (CCS) is one of the many suggested climate mitigation actions required to reduce the temperature increase the world otherwise will see. Many argue that CCS is an excuse to continue using fossil fuels. However, CCS may be the only option to reduce the CO_2_ emission from waste-to-energy plants and parts of the process industry, where the formation of CO_2_ cannot be avoided. Postcombustion CO_2_ capture using aqueous amine solvents is a mature technology with full-scale implementation at power stations.

The postcombustion CO_2_ capture technology is based on chemical reactions between a solvent and CO_2_. The solvent is often an aqueous solution of one or several amines. It is hard to have complete control over all chemical reactions happening in this complex system. It is, therefore, not unexpected that also unwanted reactions occur. In CO_2_ capture, the primary reaction is the reaction between amine and CO_2_, but other unwanted reactions form degradation products. These degradation reactions can either be reversible or irreversible. Most of the degradation compounds result in the solvent’s CO_2_ capacity loss. In many cases, solvent makeup and solvent reclaiming are used to maintain the capacity of the solvent. However, they increase the operational cost.

Knowing the identity of the unwanted products is important from an environmental and operational perspective. The degradation products formed contain various functional groups, such as acids, amines, aldehydes, amides, nitrosamine, and urea. Some of them require monitoring either from environmental or health aspects. Examples are volatile compounds such as ammonia, smaller alkylamine, and aldehydes (methylamine, ethylamine, formaldehyde, acetaldehyde, etc.) and compounds such as nitrosamine, which are known carcinogens.

The most known and studied amine is monoethanolamine (MEA), with extensive work done on the characterization and quantification of degradation compounds. The characterization and quantification of degradation compounds are time-consuming, requiring advanced analytical instrumentation and standards of the specific degradation compounds. These standards must be either commercially available or synthesized. The knowledge gained from the thorough characterization of degradation compounds in MEA and the pathways suggested for their formation are valuable and, in many cases, transferable to other amines, as long as the amine’s molecular structure is considered. Thus, degradation compounds could be divided into general and solvent-specific degradation compounds. Ammonia, smaller alkylamines, aldehydes, and some acids can be regarded as general degradation compounds. Solvent-specific degradation compounds include larger amines (e.g., diamine, methylated/alkylated amine, and demethylated/alkylated amine), cyclic structures (such as imidazole, piperazinone, oxazolidinone, and imidazolidinone), amino acids (some acids), amide, nitrosamine, nitramine, and so forth.

For postcombustion CO_2_ capture technology, the solvent technology is often proprietary, and only a few open solvents, such as MEA, piperazine (Pz), and the CESAR 1 [blend of 2-amino-2-methyl-1-propanol (AMP) and Pz] are available for commercial operation. In the EU project, HiperCAP, a solvent forming bicarbonate to a larger extent than carbamate, was developed.^1−4^ In the project, 15 amines were investigated. The evaluation of the candidates was based on experimental cyclic capacity, p*K*_a_ measurements, and solvent behavior (foaming, precipitation tendencies, and viscosity).^5^ Based on the performance, two tested candidates, 2-piperidineethanol (2-PPE) and 1-(2-hydroxyethyl)pyrrolidine (1-(2HE)PRLD), were characterized further by measuring the vapor–liquid equilibrium from 40 to 120 °C, density and viscosity from 20 to 80 °C, and thermal and oxidative degradation.^6^ The low oxidative stability of 2-PPE made this less relevant as a CO_2_ capture solvent. The amines forming mainly/more bicarbonate often have the drawback of a slow reaction with CO_2_ and are, therefore, often combined with a primary amine (a promoter). Several promoters were tested, and the blend of 40 wt % 1-(2HE)PRLD and 15 wt % 3A1P(3), with a similar cyclic capacity to CESAR 1, was chosen for further studies. The work has continued in the REALISE project,^7^ with studies on solvent characterization, composition optimization, degradation studies, piloting, and demonstration.

Regarding degradation, some thermal and oxidative degradation data for the primary amine, 3A1P, is available. For oxidative degradation, data is available at both high oxygen concentration (96–98% O_2_) in the presence of iron^8,9^ and low (21% O_2_) oxygen concentration without iron.^10^ Without metals present and a low concentration of oxygen added, 3A1P showed similar stability as MEA. However, this was not the case at high oxygen concentrations and iron present. In this case, 3A1P had higher stability than MEA. Several thermal degradation studies investigating chemical stability in the presence of CO_2_ and temperatures between 135 and 165 °C have also shown that 3A1P is more stable than MEA.^11−15^

Several degradation studies have focused on blends and, to some extent, on the interaction between the amines in the blends when it comes to degradation compounds. Du, Wang, and Rochelle^16^ conducted thermal degradation experiments for 36 Pz blends, including imidazoles, cyclic and long-chain diamines, tertiary amines, hindered amines, amino acids, and ether amines. One of the tertiary amines investigated was 1-(2-hydroxyethyl)piperidine (HEPD/1-(2HE)PP), which is similar to 1-(2HE)PRLD since both have the same substituent on the nitrogen atom in the ring, but HEPD is a 6-membered ring, while 1-(2HE)PRLD is a five-membered ring.

This work focuses on the chemical stability of a blend of 1-(2HE)PRLD and 3-amino-1-propanol (3A1P). Data for both the single system of 1-(2HE)PRLD and blends under conditions simulating the absorber (oxidative degradation), desorber (thermal degradation), and the process as a whole through circulating the solvent between conditions relevant for the absorber to the stripper will be presented.

Degradation is a slow process; therefore, lab experiments are typically designed to generate accelerated degradation. Factors that accelerate degradation are oxygen concentration, temperature, and the addition of metals (often iron). In this work, thermal degradation experiments were conducted at 135 °C in the presence of CO_2_. The oxidative degradation experiments were conducted in two different setups and two different conditions [medium (21%) and high (96%) oxygen concentration, with and without iron added]. The influence of promoter concentration was investigated under highly accelerated conditions where a high oxygen concentration (96%) was combined with iron (0.5 mM). The connection between thermal and oxidative degradation was investigated using a cyclic degradation setup where the solvent circulated between low and higher temperatures and was allowed to make contact with a synthetic blend gas of N_2_, O_2_, CO_2_, and NOx on the low-temperature side. Previously, these cycled degradation experiments have been shown to give a more pilot-like degradation profile than batch experiments.^17,18^ In parallel to the experiments, work was done on identifying degradation compounds in the blends. The most comprehensive characterization of solvent samples was done during the cyclic degradation experiment performed last. These solvent samples were analyzed for 44 different compounds using liquid chromatography–mass spectrometry (LC–MS).

## 2 Experimental Procedures

The chemicals used to prepare amine solutions are given in Table 1, while Table 4 provides an overview of the experiments and concentrations of the solutions used. All chemicals were used without further purification. Aqueous amine solutions were prepared gravimetrically. For all experiments, a CO_2_-loaded amine solution was prepared by bubbling CO_2_ through the aqueous amine solutions until the desired weight of CO_2_ was reached. The amine concentration and CO_2_ content were then confirmed by analyzing the amine and CO_2_ content in the solutions using titration (amine concentration) and total organic carbon analyses (CO_2_ content).

**Table 1:**
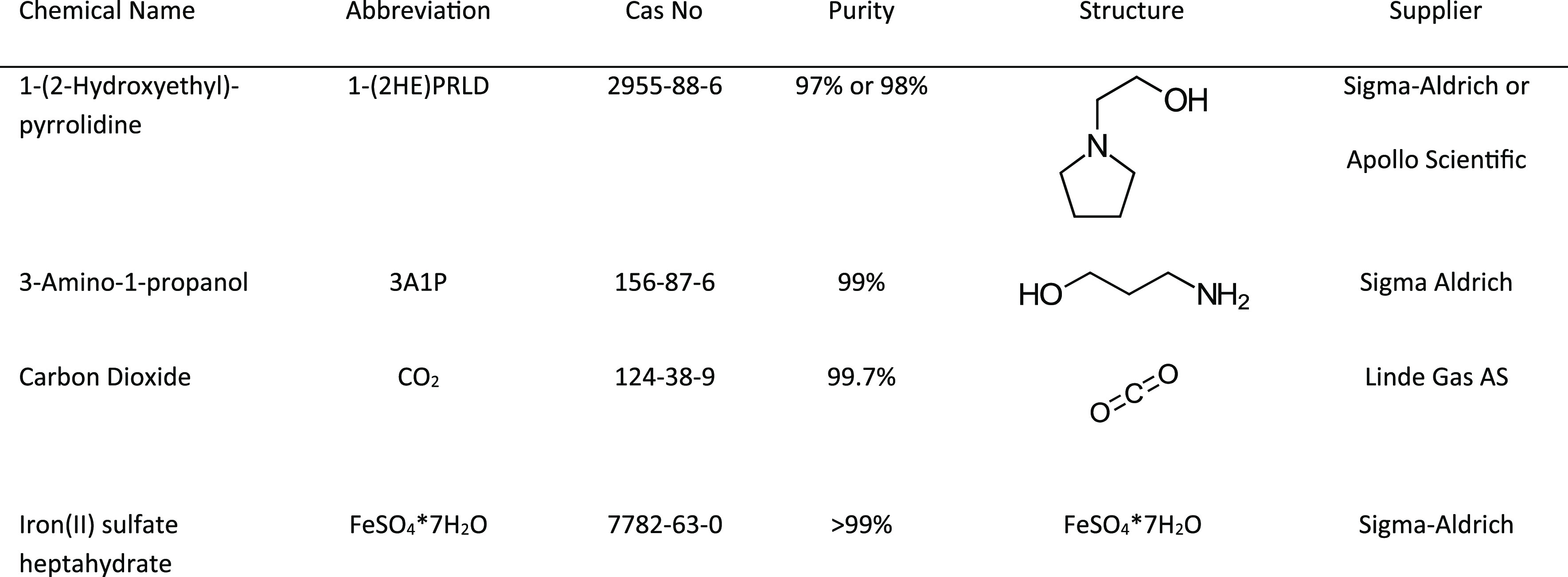
Chemicals Used to Prepare Solutions

### 2.1 Degradation Setups

#### 2.1.1 Thermal Degradation Experiments

Thermal degradation experiments were conducted as described by Lepaumier, Grimstvedt, Vernstad, Zahlsen, and Svendsen^19^ using stainless-steel-tube cells (316SS, OD = 1.22, thickness = 1.7 mm) of about 27 cm^3^ total volume with a Swagelok valve. The cell was flushed with N_2_ before adding 15 cm^3^ of the CO_2_-loaded amine solution. The top of the cell was flushed with N_2_ (to remove air) before the valve was closed. The cells were placed in a convection oven at 135 °C; one cell was drawn every week, and the last cell was taken after 5 weeks.

#### 2.1.2 Oxidative Degradation Experiments

Oxidative degradation experiments were performed using two different setups. The experimental conditions are presented in Table 2. Setup 1^20,21^ and setup 2^7^ have been presented in detail elsewhere with the experimental procedures. Thus, the experimental methods are only shortly presented here. For both setups, a CO_2_-loaded amine solution was used. All experiments were run for 21 days. Setup 1 is an open-batch glass reactor (liquid volume of 1 L) where a synthetic wet gas of air (98%) and CO_2_ (2%) was sparged into the solution continuously throughout the experiment. A recycle loop maintained a gas blend circulation rate of about 50 L/h. Two condensers were placed on the ’reactor’s outlet to reduce the water evaporation. In setup 2, the experiments were conducted in open and water-bath heated jacketed glass reactors (liquid volume of 200 mL) with a gas mixture of 96% O_2_ and 4% CO_2_ sparged into the solution throughout the experiment. Each reactor was connected to a condenser, reducing the loss of volatile components (mostly water) from the reactor. A magnetic stirrer provided stirring. During the experiments with setups 1 and 2, solvent samples from the reactors were taken regularly and analyzed.

**Table 2:** Overview of the Conditions during Oxidative Degradation Experiments in Setups 1 and 2

setup	reference for description	reactor *T* °C	liquid volume (L)	gas composition	iron added (mM)
setup 1	Vevelstad et al.^22^	55	1	21% O_2_,2% CO_2_, N_2_ rest	no
setup 2	Buvik et al.^8^	60	0.2	96% O_2_,4% CO_2_	0.5

#### 2.1.3 Degradation in the Cyclic Degradation Setup (SDR)

The solvent degradation rig (SDR) is designed to investigate degradation under more realistic conditions. SDR allows investigations where oxidative degradation, thermal degradation, and the effect of other impurities (NOx) are studied and where connections between them can be seen. A detailed description of the equipment, including a picture of the setup, can be found elsewhere.^17,18^ The solvent is circulated between an absorber and a desorber in the setup. The rig is designed for studying the degradation and mapping of degradation compounds.^17,18^ However, unlike in an industrial plant, the CO_2_ gas from the desorber is fed back to the absorber, meaning that the rig is semiclosed and that there will be a build-up of volatile components such as ammonia in the solvent. Thus, the rig only gives a qualitative picture of the emission.

The solvent stability can be stressed by increasing the desorber temperature or increasing the NOx concentration to investigate the robustness of the solvent regarding forming nitrosamine. The changes in the operating conditions are made stepwise and sequentially. This is because degradation is a slow process, and to quantify degradation compounds, they need to be above the lower limit of quantification (LOQ). The process conditions used in this work are given in Table 3. These are the same conditions as used in previous works.^23^

**Table 3:** Main Process Conditions during the SDR Campaign

	CO_2_ concentration (vol %)	oxygen concentration (vol %)	absorber *T* (°C)	desorber *T* (°C)	NOx (ppm)	time (week)
standard conditions	3	12	∼40–50	120	5	1–3
high desorber *T*	3	12	∼40–50	140	5	4
high NOx	3	12	∼40–50	120	50	5

### 2.2 Analysis

The samples from the different setups were analyzed using various analytical techniques described here. Table 4 gives an overview of which analyses were conducted at which experiments.

**Table 4:** Overview of the Experiments and Analyses Conducted

										anion/acids		degr.cpd. (LC–MS)						
studying	setup	solvent composition—wt % (A = 1(2HE)PRDL, B = 3A1P)	density	CO_2_	H_2_O	HSS	nitrogen	amine titration	LC–MS	IC-anion	LC–MS	ammonia and alkylamine	nitrosamine	various degr.cpd.	aldehyde + acetone	total nitrosamine	metals	ref
thermal degr.	cells	40 A + 15 B	x	X				x	x								k	this work
thermal degr.	cells	30 A		X				x	x								k	(1)
thermal degr.	cells	30 B		X					x								l	(11)
oxidative degr.	R1	30 B	x	X		x	Kjeldahl	x	x	a		d					m	(24)
oxidative degr.	R1	30 A	x	X	x		Kjeldahl	x	x			c	f	h				(1)
oxidative degr.	R1	40 A + 15 B	x	X	x		Kjeldahl	x	x			c	f	h				this work
oxidative degr.	R2	40 A		x				x	x		X			i				this work
oxidative degr.	R2	40 A + 5 B		x				x	x		X			i^,^^j^				this work
oxidative degr.	R2	40 A + 15 B		x				x	x		X			i^,^^j^				this work
oxidative degr.	R2	40 A + 20 B		x				x	x		X			i^,^^j^				this work
process degr.	SDR	40 A + 15 B	x	x	x	x	TN	x	x	b	X	e	g	i^,^^j^	x	x	n	this work

a Nitrite, nitrate, formate, oxalate, and sulfate.

b Formate and oxalate.

c Ammonia.

d Ammonia, methylamine, dimethylamine, ethylamine, and diethylamine.

e Ammonia, methylamine, dimethylamine, ethylamine, diethylamine, ethylmethylamine, propylamine, and dipropylamine.

f NPYR.

g NDELA, NDMA, NDEA, NPIP, NMEA, NPYR, NMOR, NDPA, NDBA, nitroso-*N*-methyl-AP, and NOXZN.

h Pyrrolidine.

i Pyrrolidine and 3-Mpy.

j 3-(Methylamino)-1-propanol (methyl-AP), 1,3-oxazinan-2-one (OZN), *N*,*N*′-bis(3-hydroxypropyl)-urea (AP-urea), *N*-(3-hydroxypropyl)-β-alanine (HPAla), *N*-(3-hydroxypropyl)-glycine (HPGly), *N*-(3-hydroxypropyl)-formamide (HPF), tetrahydro-1-(3-hydroxypropyl)-2(1H)-pyrimidinone (tHHPP), and 3-[(3-aminopropyl)amino]-1-propanol (APAP).

k Iron, chromium, and nickel.

l Vanadium, chromium, iron, nickel, and molybdenum.

m Sulfur.

n Iron, nickel, chromium, copper, sulfur, Zinc, barium, vanadium, sodium, and aluminum.

#### 2.2.1 Wet Chemistry and “Standard” Methods

The total amine concentration was determined by acid/base titration (0.1 M H_2_SO_4_), CO_2_ with a total inorganic carbon/total organic carbon (TIC/TOC) analyzer, H_2_O with Karl Fischer titration, total nitrogen by oxidative catalytic combustion and chemiluminescence detection (Shimadzu TOC-L CPH TNM-L) or total organic nitrogen using the Kjeldahl method.^25^ Density was measured using a densitometer (Mettler-Toledo CM40) or gravimetrically when a little sample volume was available. Finally, heat-stable salts (HSSs) were measured using a wet chemistry method based on ion exchange followed by titration with NaOH and metals by inductively coupled plasma mass spectrometry (ICP-MS).

#### 2.2.2 Liquid Chromatography

Two chromatographic techniques were used to quantify the solvent amines and the degradation components. Formate and oxalate were analyzed by an external laboratory using ion chromatography. The samples were diluted with deionized water (18.2 MΩ) and analyzed by anion chromatography, IC-EDC, a Dionex ICS 3000 system, 25 μL of loop, and IonPac AG/AS11HC guard and separation columns. A gradient method from 2 to 30 mM was used for the elution of the organic acid anions. External calibration curves were used for comparison. Typical uncertainty was within ±20% rel.

The rest of the components, including the solvent amines, were analyzed using LC–MSMS. Quantitative analysis using LC–MSMS technology requires fulfillment of the following four criteria, chromatographic separation with specific retention time (of certified reference material), molecular-ion formation related to molecular weight (e.g., M–H^+^ and M–NH_4_^+^), specific fragment-ion formation (collision-induced dissociation in collision cell at a specific set of voltages), and the ratio between formed fragments. These criteria are also used in forensic medicine. The instrumentation used was an Agilent Technologies 1290 Infinity LC system coupled with Agilent Technologies 6495 Triple Quad MS detector. More details regarding the column, mobile phase, ion source, and if derivatization has been used are described in Table 5. An internal isotope-labeled standard was used for the following components: 3A1P (AP-d4), ammonia, MA, DMA, EA, DiEA, propylamine, ethylmethylamine, dipropylamine, formaldehyde, acetaldehyde, acetone, formic acid, acetic acid, lactic acid, isobutyric acid, NDMA, NMEA, NPYR, NMOR, NDELA, NDEA, NPIP, NDPA, NDBA, and DMA-NO_2_. Components analyzed by the various methods of LC–MS are listed in Table 6.

**Table 5:** Overview Conditions (Ion Source, Column, Mobile Phase, and if Derivatization Has Been Used) for the Different Analyses

component	ion source	column	mobile phase	derivatization
amine	APCI	Ascentis Express phenyl-hexyl,2.7 μm HPLC column	0.1% trifluoroacetic acid (A), methanol (B), gradient	no
degradation mix	ESI	Discovery HS F5 HPLC column	0.1% formic acid (A), methanol (B), gradient	no
ammonia and alkylamine	ESI	Ascentis Express C18 column	0.1% formic acid (A), acetonitrile (B), gradient	dansyl chloride
aldehyde and acetone	ESI	Ascentis Express C8 column	0.1% ammonium acetate (A), acetonitrile (B), isocratic	dinitrophenylhydrazine
organic acids	ESI	Waters Acquity HSS-T3(15 × 2.1 mm)	0.05% acetic acid (A), acetonitrile (B), gradient	3-nitrophenylhydrazine
nitrosamine	APCI	Ascentis Express phenyl-hexyl,2.7 μm HPLC column	0.1% formic acid (A), acetonitrile (B), gradient	no
nitramine	ESI	Agilent Eclipse plus C18 RRHD 1.8 μm(2.1 × 50 mm)	0.1% ammonium acetate (A), isocratic	no

**Table 6:** Overview of Components Analyzed Using LC–MS Divided into Groups and Described in Which Experiments These Components Have Been Used

group	name	CAS	abb.	experiments where it has been analyzed for
solvent amine	3-amino-1-propanol	156-87-6	3A1P	SDR, oxidative, thermal
	1-(2-hydroxyethyl)pyrrolidine	2955-88-6	1-(2HE)PRLD	SDR, oxidative, thermal
degradation mix	3-(methylamino)-1-propanol	42055-15-2	methyl-AP	SDR, oxidative
	1,3-oxazinan-2-one/tetrahydro-2*H*-1,3-oxazin-2-one	5259-97-2	OZN	SDR, oxidative
	*N*,*N*′-bis(3-hydroxypropyl)-urea	71466-11-0	AP-urea	SDR, oxidative
	pyrrolidine	123-75-1	pyrrolidine	SDR, oxidative, thermal
	3-methyl-pyridine	108-99-6	3-Mpy	SDR, oxidative
	*N*-(3-hydroxypropyl)-β-alanine	55937-35-4	HPAla	SDR, oxidative
	*N*-(3-hydroxypropyl)-glycine	100747-20-4	HPGly	SDR, oxidative
	*N*-(3-hydroxypropyl)-formamide	49807-74-1	HPF	SDR, oxidative
	tetrahydro-1-(3-hydroxypropyl)-2(1*H*)-pyrimidinone	670227-88-0	tHHPP	SDR, oxidative
	3-[(3-aminopropyl)amino]-1-propanol	40226-15-1	APAP	SDR, oxidative
ammonia and alkylamine	ammonia	7664-41-7	NH_3_	SDR
	methylamine	74-89-5	MA	SDR
	ethylamine	75-04-7	EA	SDR
	propylamine	107-10-8		SDR
	dimethylamine	124-40-3	DMA	SDR
	ethylmethylamine	624-78-2		SDR
	diethylamine	109-89-7	DiEA	SDR
	dipropylamine	142-84-7		SDR
Aldehyde and acetone	formaldehyde	50-00-0		SDR
	acetaldehyde	75-07-0		SDR
	acetone	67-64-1		SDR
organic acids	glycolic acid	79-14-1		SDR
	formic acid	64-18-6		SDR
	propionic acid	79-09-4		SDR
	isobutyric acid	79-31-2		SDR
	lactic acid	50-21-5		SDR
	acetic acid	64-19-7		SDR
	*N*-butyric acid	107-92-6		SDR
	glyoxylic acid	298-12-4		SDR
nitrosamine	3-(methylnitrosoamino)-1-propanol	70415-59-7	nitroso-*N*-methylAP	SDR
	*N*-methyl-*N*-nitroso-methanamine	62-75-9	NDMA	SDR
	*N*-methyl-*N*-nitroso-ethanamine	10595-95-6	NMEA	SDR
	1-nitroso-pyrrolidine	930-55-2	NPYR	SDR
	4-nitroso-morpholine	59-89-2	NMOR	SDR
	2,’2′-(nitrosoimino)bis-ethanol	1116-54-7	NDELA	SDR
	*N*-ethyl-*N*-nitroso-ethanamine	55-18-5	NDEA	SDR
	1-nitroso-piperidine	100-75-4	NPIP	SDR
	*N*-nitroso-*N*-propyl-1-propanamine	621-64-7	NDPA	SDR
	*N*-butyl-*N*-nitroso-1-butanamine	924-16-3	NDBA	SDR
	tetrahydro-3-nitroso-2*H*-1,3-oxazine	35627-29-3	NOXZN	SDR
nitramine	dimethylnitramine	4164-28-7	DMA-NO_2_	SDR

Additionally, total nitrosamine was measured using a group method developed at SINTEF. The method is based on principles published by Wang et al., 2005,^26^ where nitrosamine is measured after chemical denitrosation and subsequent chemiluminescence detection. Total nitrosamines are measured as released NO gas (from the nitroso group of the nitrosamine) using a the nitrogen chemiluminescence detector (NCD) after treatment of the sample with CuCl and HCl. The instrument used was Agilent GC 7890A coupled with an Agilent Technologies 255 NCD, Agilent Technologies G6641A Dual Plasma Controller, and Agilent 7697A Headspace Sampler. A J&W GS-GasPro GC column, 60 m, 0.32 mm, was used. The instrumental LOQ was 0.68 μmol/L. The typical uncertainty of the method was 10% (matrix-related).

### 2.3 Data Treatment and Experimental and Analytical Uncertainty

One of the challenges in evaluating data from different experimental setups and campaigns is that different information is available, making comparing data on the same grounds challenging. The data given could be based on many assumptions, and the experimental and analytical uncertainty is often not given. For example, the concentration given for different analytes can be on a mass or volume basis. When using mass, the effect of the CO_2_ loading must be taken into account as CO_2_ adds weight to the solution, but it is considered not to add volume. However, moving from mass to volume-based concentrations requires the measurement of density, something that sometimes is lacking. Another challenge in open and semiclosed degradation systems is water balance, as some water will leave with the gas. Often, water is measured, or an internal standard is added to adjust the water concentration in the end.

Thus, it is essential to consider experimental and analytical uncertainties when evaluating degradation data. One typical challenge is the calculation of the rate of degradation based on amine loss (i.e., the difference between two amine concentrations), where uncertainty in analyzed amine concentration could be as high as 5% relative (depending on which analytical technique is used), giving a high relative uncertainty of the calculated rate when the concentration difference is small. In addition, the total uncertainties will be influenced by the uncertainties during experiments (such as water balance) and sampling.

Due to the difference in both water and CO_2_ content in the samples, some corrections are applied to the results from the SDR rig. We have used a correction for both water and CO_2_ as given in eq 1
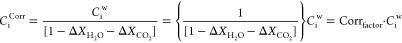
1where
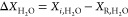
2
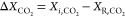
3

In the equations, *C*_i_^w^ is the analyzed concentration on a weight basis (i.e., mg/kg), Δ*X*_H_2_O_ is the difference in the weight fraction of water between the actual sample and a reference, and Δ*X*_CO_2__ is the difference in the weight fraction of CO_2_ between the actual sample and a reference.

Applied corrections have to be included in the uncertainty calculation. For example, for the correction above, the uncertainty could be split into uncertainty in the correction factor and the measured concentration (*C*_i_^w^). More details regarding the uncertainty calculations can be found in Supporting Information Chapter 3.1. No corrections were made to the data from setups 1 and 2.

## 3 Results and Discussion

### 3.1 Thermal Degradation

**Figure 1:**
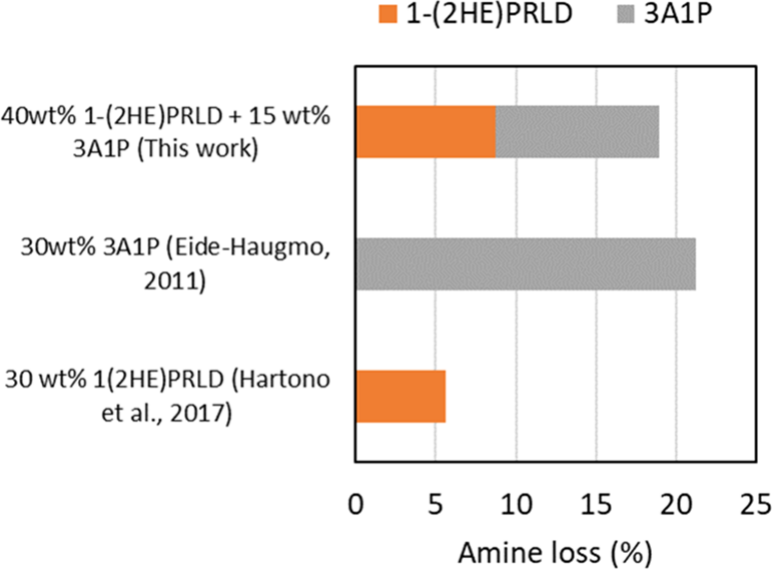
Amine loss (mole based %) for thermal degradation (with CO_2_) for single amines as well as the blend (this work: *C*_A,0_ = 5.48 α = 0.4 mol CO_2_/mol amine, Eide-Haugmo 2011:^11^*C*_A,0_ = 4.00 α = 0.5 and Hartono et al. 2017:^1^*C*_A,0_ = 2.29 α = 0.4).

The blend of 1-(2HE)PRLD and 3A1P was tested for thermal degradation in the presence of CO_2_ at 135 °C for 5 weeks. Figure 1 compares the amine loss of the blend with literature data for the single amine systems.^1,11^ The initial amine and CO_2_ concentration varied between the experiments impacting the results; for example, the higher loading of CO_2_ in the single 3A1P experiment is expected to give higher amine loss.^27^ The same has been seen for aqueous MEA during similar experiments. In the reported results with MEA at concentrations of 4–5 mol/L, the thermal degradation was around 40–45% at loading 0.4 mol CO_2_/mol amine and 56% at loading 0.5 mol CO_2_/mol amine.^11,28^

As mentioned above, the initial amine concentration and CO_2_ concentration vary in all the experiments in Figure 1, leading into that a direct comparison is tricky. However, the respective loss of 1-(2HE)PRLD and 3A1P in the blend is almost identical. This may indicate that the presence of 1-(2HE)PRLD is favorable for 3A1P. In work by Eide-Haugmo,^9^ the chemical stability of tertiary amines seemed to increase with the size of the substituents, meaning tertiary amines with methyl substituents (e.g., DMMEA—108-01-0, MDEA—105-59-9) were more easily degradable than tertiary amines with ethyl or ethanol substituents (DEEA—100-37-8 and TEA—102-71-6).^11^ The loss of 1-(2HE)PRLD alone or in the blend varies between 6 and 10%, which is in the same order as for TEA (10%).^11^

### 3.2 Oxidative Degradation

**Figure 2:**
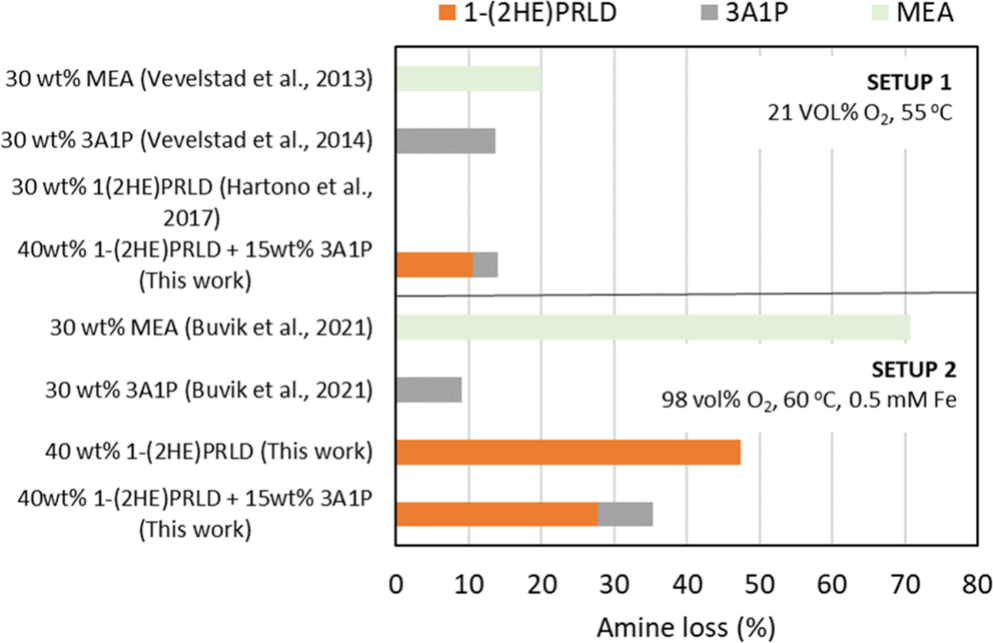
Amine loss (mole based %) for single amine solutions and the blend in setups 1 and 2 with different temperatures, oxygen, and iron concentrations. 30 wt % MEA—Vevelstad et al. 2013,^29^ 30 wt % 3A1P—Vevelstad et al. 2014,^24^ 30 wt % 1(2HE)PRLD—Hartono et al. 2017,^1^ and 30 wt % MEA and 30 wt % 3A1P—Buvik et al. 2021.^8^

Oxidative degradation experiments were conducted with variations in the conditions (medium or high oxygen concentration and the presence of metal), which is, therefore, causing significant differences in the results. The oxygen concentration in the flue gas is typically between 4 and 15%, but metals such as iron will always be present in a solvent-based CO_2_ absorption plant. An overview of the results for the single amine systems and the blend at different process conditions is given in Figure 2. All experiments lasted 21 days in a glass reactor with gas bubbled (O_2_, CO_2_, and N_2_) through an amine solution preloaded with CO_2_ (α = 0.4 mol CO_2_/mol amine). For the single system, the available literature data is also plotted^1,8,24,29^ in Figure 2.

For MEA, increased amine loss is reported with increasing oxygen concentration, as expected.^22,29^ Furthermore, iron in the experiment with 96% O_2_ is also expected to reduce chemical stability.^27,30,31^ However, the different experimental conditions show considerable variation between the single system 3A1P and 1-(2HE)PRLD. For 3A1P, higher chemical stability is observed for the experiment with iron and high oxygen concentration, while the opposite was observed for 1-(2HE)PRLD. There is no clear explanation for this. However, as discussed for thermal degradation experiments, 1-(2HE)PRLD is more stable than other tertiary amines because of the ethanol substituent. At the same time, the increased oxygen concentration might impact the decomposition of the molecule. For the blend, iron and high oxygen concentration seem to reduce the chemical stability with a factor of 2.5 under the conditions investigated in this work.

**Figure 3:**
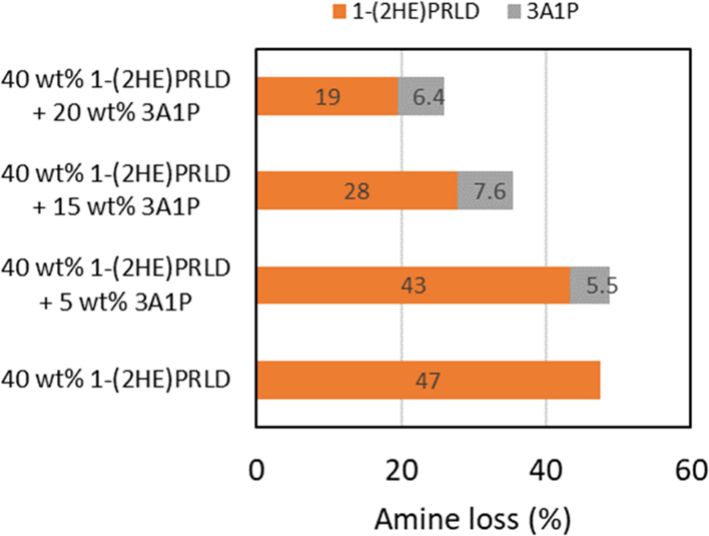
Amine loss (mole based %) for the blend with a constant concentration of 40 wt %, while the concentration of 3A1P varies from 0 to 20 wt %.

The promoter’s concentration effect on chemical stability was investigated under the harshest conditions (96% O_2_, 60 °C, 0.5 mM Fe). The amine losses for these experiments are shown in Figure 3. Under the tested conditions (high oxygen concentration and presence of iron), the chemical stability of the blend increases with increasing 3A1P concentration as long as the 3A1P concentration is higher than 5 wt %. The loss of 3A1P is relatively similar for all concentrations, including 30 wt % 3A1P in Figure 2. To our knowledge, the promoter concentration effect on oxidative degradation in other blends has not been investigated.

#### 3.2.1 Degradation Compounds in Experiments with 96% O_2_ (Setup 2)

**Figure 4:**
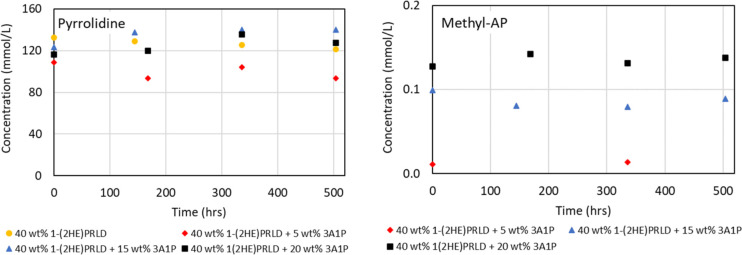
Concentration (mmol/L) of pyrrolidine and methyl-AP as a function of time (h) for the oxidative degradation experiments at 96% O_2_, 0.5 mMFe, 60 °C.

The experiments investigating the promoter concentration were analyzed for carboxylic acids (glycolic, formic, acetic, propionic, isobutyric, *N*-butyric, lactic, and glyoxylic acid), amines (pyrrolidine, APAP, and methyl-AP), amide of 3A1P (HPF), urea (AP-urea), ring structures (OZN, 3-MPy, and tHHPP), amino acids (HPGly and HPAla). The following components were not observed over the LOQ in any of the experiments: acids (isobutyric, *N*-butyric, lactic, and glyoxylic acid), 3-Mpy, and tHHPP. The found compounds can be divided into impurities in the solvent and degradation compounds depending on if they are present in the start sample and how the concentration changes throughout the experiments. Pyrrolidine and methyl-AP seem to be impurities under these conditions as the concentration (mmol/L) is constant as a function of time, as shown in Figure 4. The concentration of pyrrolidine is relatively constant for all experiments. Methyl-AP’s concentration is relatively stable over time but increases with the initial 3A1P concentration in the experiments. Later, it will be shown that these components could also be degradation compounds. However, this does not seem to be the case under purely oxidative degradation conditions at low temperatures.

**Figure 5:**
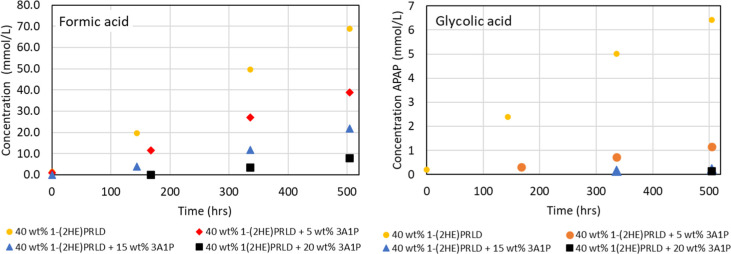
Concentration (mmol/L) of formic and glycolic acid as a function of time (h).

**Figure 6:**
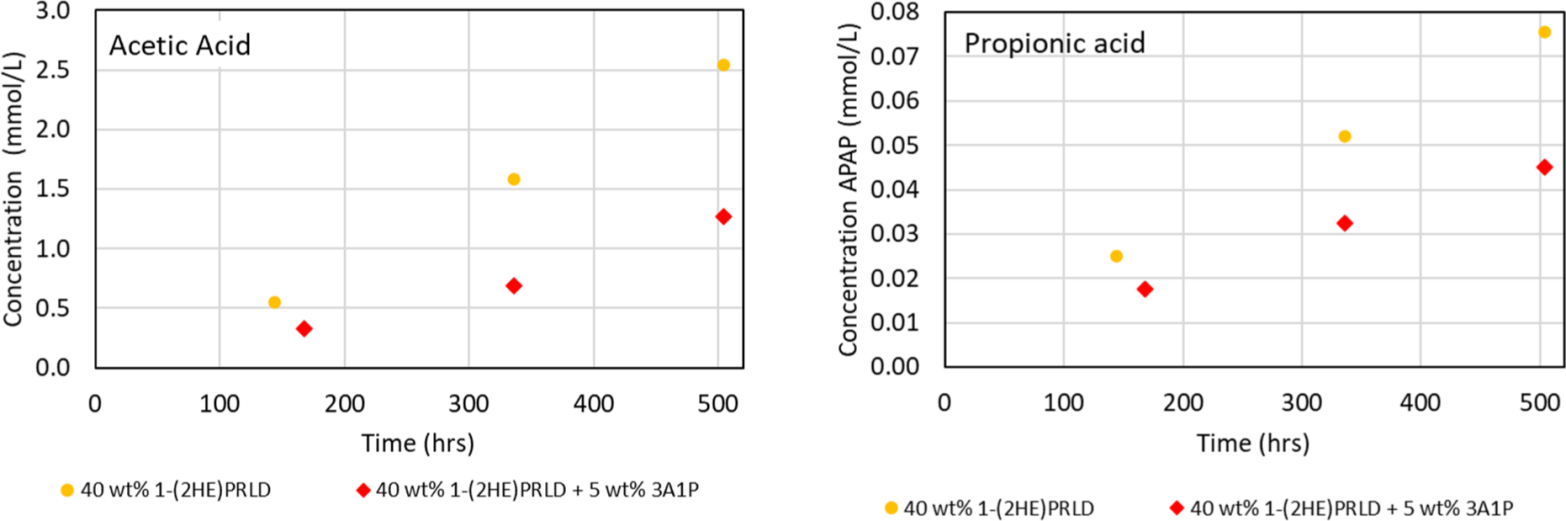
Concentration (mmol/L) of acetic and propionic acid as a function of time (h). Acetic and propionic acid were below the LOQ for 40 wt % 1-(2HE)PRLD + 15 wt % 3A1P and 40 wt % 1-(2HE)PRLD + 20 wt % 3A1P.

The concentration of formic, glycolic, acetic, and propionic acid as a function of time (h) are shown in Figures 5 and 6. The build-up of acids increases with the decreased stability of the blend. It is also clear that the concentration increases with decreasing overall amine concentration or increasing promoter concentration. Loading is kept constant, meaning that CO_2_ concentration increases with the initial amine concentration. Part of the explanation for higher stability at higher initial amine concentration could result from lower oxygen solubility since there will be higher amounts of ionic components in the solution.^32^

**Figure 7:**
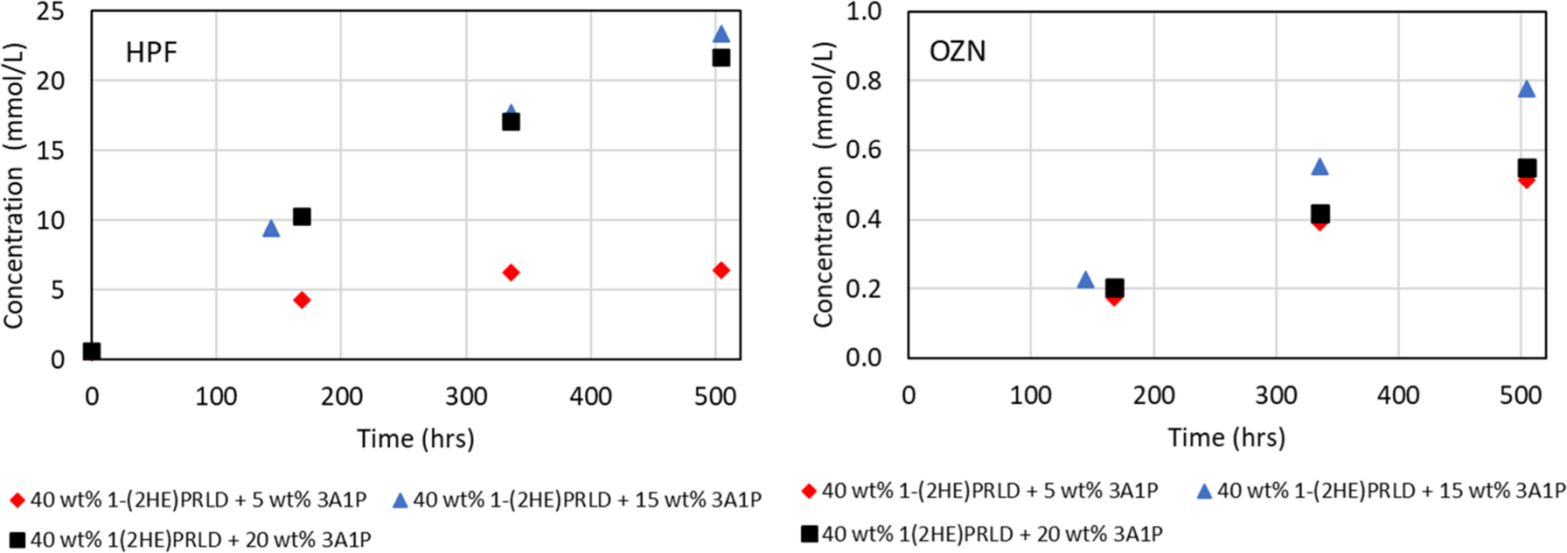
Concentration (mmol/L) of HPF and OZN as a function of time (h) for the blend with 40 wt % 1-(2HE)PRLD and either 5, 15, or 20 wt % 3A1P.

**Figure 8:**
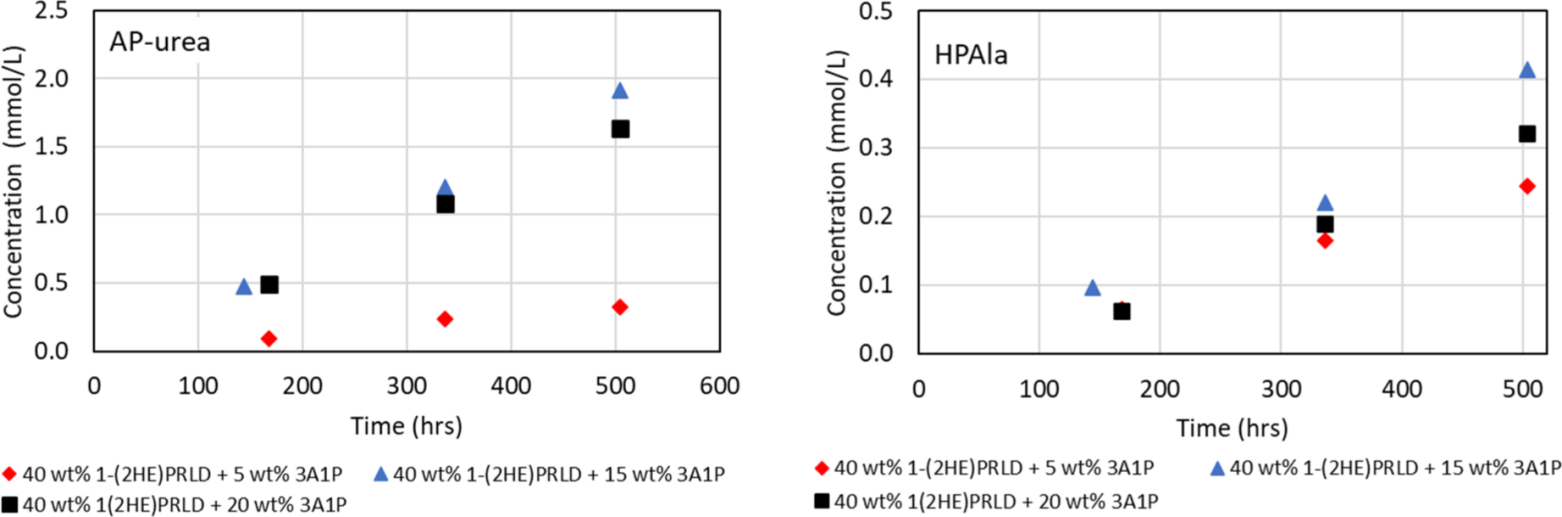
Concentration (mmol/L) of AP-urea and HPAla as a function of time (h) for the blend with 40 wt % 1-(2HE)PRLD and either 5, 15, or 20 wt % 3A1P.

**Figure 9:**
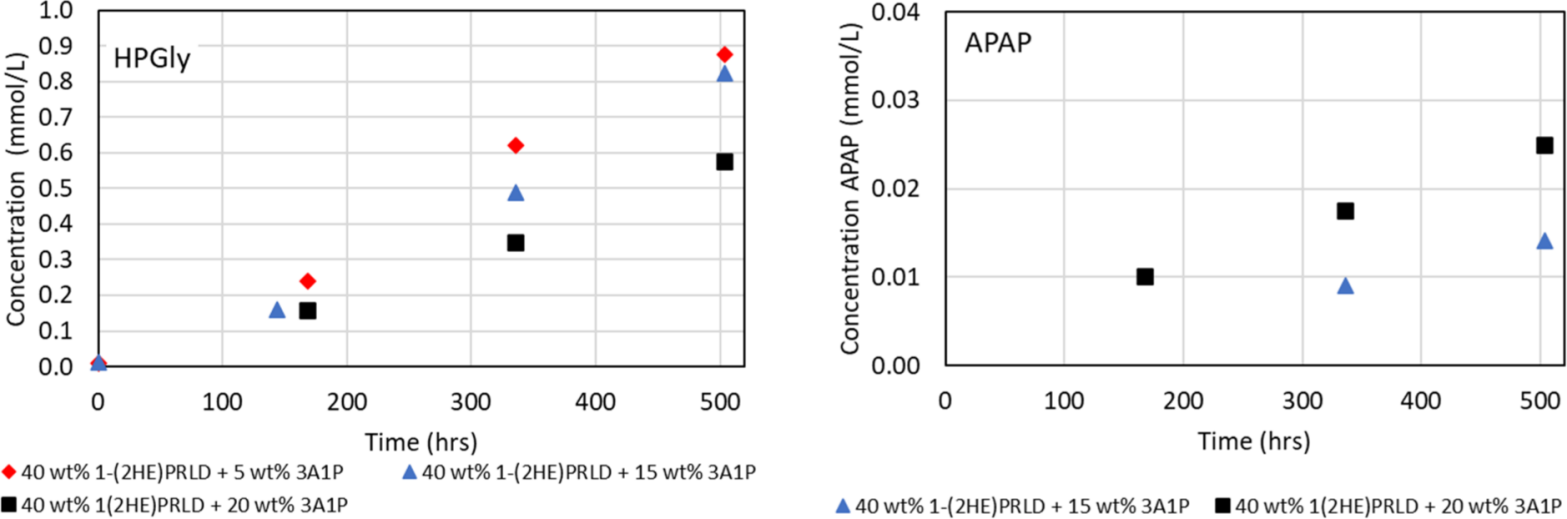
Concentration (mmol/L) of HPGly and APAP as a function of time (h) for the blend with 40 wt % 1-(2HE)PRLD and either 5, 15, or 20 wt % 3A1P. APAP was below the LOQ for 40 wt % 1-(2HE)PRLD + 5 wt % 3A1P.

Figures 7–9 show the concentration (mmol/L) as a function of time (h) for HPF, OZN, AP-urea, HPAla, APAP, and HPGly. The major component among these under these conditions was HPF. The build-up of degradation compounds shown in Figures 7 and 8 partly seems to increase with increasing 3A1P concentration for the two lowest concentrations of 3A1P (5 and 15 wt %). However, for the experiments at 20 wt %, other factors seem to have a more significant role. In general, the highest amine concentration and, therefore, the highest promoter concentration give the highest concentration of HPF, OZN, AP-urea, and HPAla. The opposite is observed for carboxylic acids in Figures 5 and 6, where a decrease in acidic components is seen at increasing amine concentrations. This is natural since the carboxylic acid and the amides are part of a reversible reaction, where the equilibrium seems to be toward the amide at a higher amine concentration and the acid at a lower amine concentration. HPF, OZN, AP-urea, and HPAla are formed through certain intermediates that are related to 3A1P or CO_2_ concentration, or both. It is, therefore, expected that experiments with the highest amine concentration and, in particular, 3A1P concentration will favor the formation of these components.

Figure 9 shows the concentration of HPGly and APAP (mmol/L) as a function of time (h) for the blends containing 3A1P. APAP shows behavior where a higher 3A1P concentration increases the formation. This is natural since 2 moles of 3A1P will be required for its formation. However, HPGly behaves differently. The molecular structure of the component itself, combined with these experimental results, indicates that HPGly is a product of the blend and would not be formed in systems with only one of the amine components present.

### 3.3 Cyclic SDR

The blend, 40 wt % 1-(2HE)PRLD and 15 wt % 3A1P, was also tested under more realistic conditions using a SDR, where the solvent is cycled between the absorber and the desorber. To investigate the robustness of the solvent, a test program was implemented where the first 3 weeks were under the so-called standard conditions (an oxygen concentration of 12%, a desorber temperature of 120 °C, and a NOx concentration of 5 ppm) as shown in Table 3. After 3 weeks, the desorber temperature was increased to 140 °C, and in the last week, the desorber temperature was returned to 120 °C, while the NOx concentration was increased to 50 ppm.

**Figure 10:**
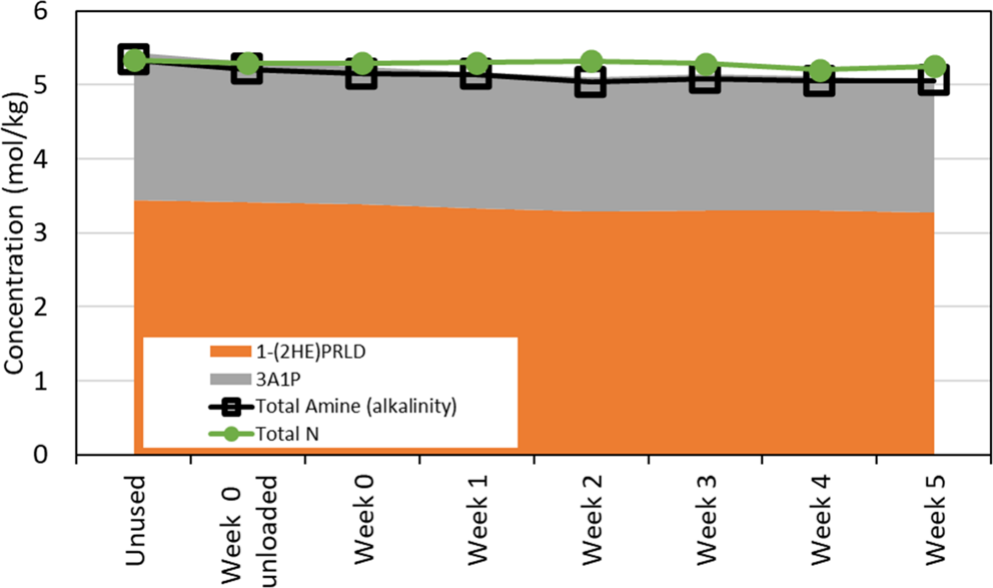
Concentration (mol/kg) of 1-(2HE)PRLD, 3A1P, alkalinity, and total nitrogen in the lean samples as a function of time (week)—the data has been corrected for water and CO_2_.

The lean samples were analyzed for alkalinity (titration, solvent amines + degradation compounds), solvent amines (LC–MS), and total nitrogen. Figure 10 shows that the alkalinity and solvent amines match each other, indicating that other amines formed as degradation compounds are only present in a small amount. The deviation between total nitrogen and the titration and LC–MS results shows that nitrogen-containing degradation compounds are formed without an amine functionality. A relative loss of 2% in alkalinity and 4% of amine (1-(2HE)PRLD + 3A1P) was observed. For the MEA experiment in the same setup operated under similar conditions^23^ (an initial MEA concentration of 30 wt %), the relative loss in alkalinity was 3%, and the loss in MEA around 4% (the same correction as given in eq 1 applied for both of the data sets).

#### 3.3.1 Characterization of Degradation Compounds

The solvent and condensate samples went through a thorough analytical program, specifically looking for 42 different compounds in addition to the solvent amines. 30 of these specific degradation compounds were observed in the solvent, condensate, or both samples (as shown in Supporting Information Table S21). The 12 compounds not observed over the LOQ are either not formed in this solvent or, as the build-up of degradation compounds takes time, require more time to be formed in amounts exceeding the LOQ.

**Figure 11:**
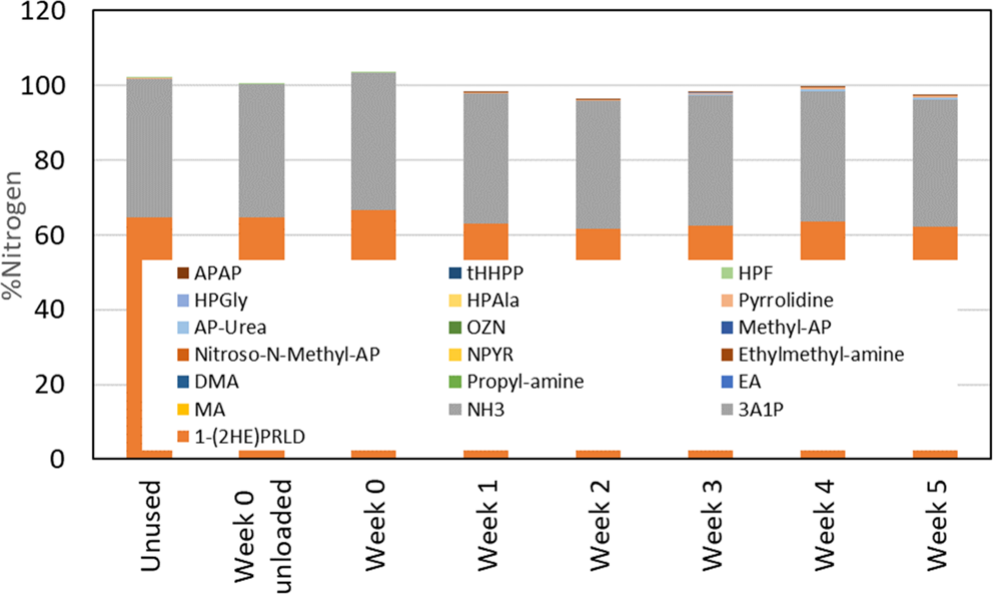
Total nitrogen balance for the solvent samples showing the solvent ’components’ contribution to the nitrogen balance.

**Figure 12:**
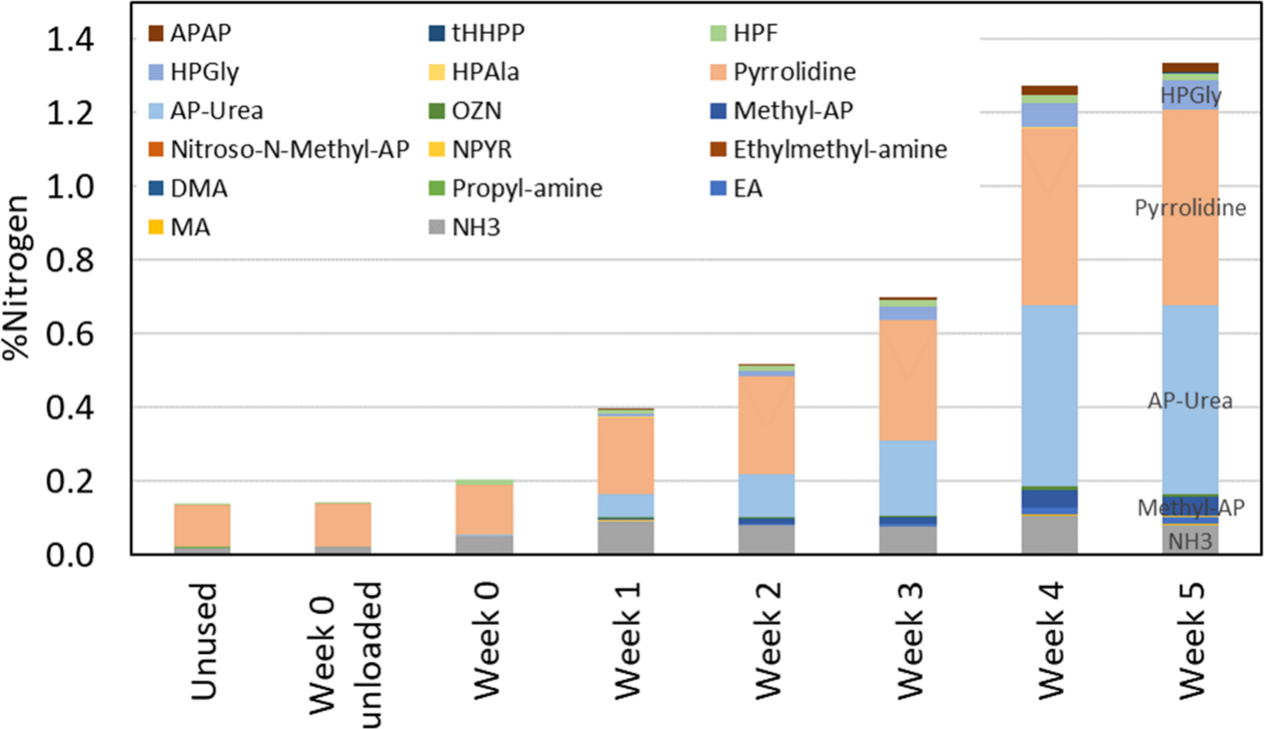
Degradation ’compounds’ contribution to the nitrogen balance.

In addition, several analytical methods were used to get an overall view of the solvent’s health. These included amine titration, total nitrogen, HSSs, and total nitrosamine. Amine titration gives the total alkalinity in the solution, including the solvent components and degradation compounds with amine functionality. In Figure 10, the results from amine titration and total nitrogen agree well with the sum of 1-(2HE)PRLD and 3A1P concentrations. There is a slight overprediction, which is not uncommon as both methods have analytical uncertainties. However, three amines are also identified as degradation compounds: APAP, methyl-AP, and pyrrolidine. These degradation compounds contribute to less than 1% of the alkalinity measured in the solution. From Figure 10, it is also clear that other degradation compounds without an amine functionality are formed (with nitrogen) since the total nitrogen in the solvent is higher than what is accounted for by amine titration. A nitrogen balance was conducted for the solvent samples by summing up nitrogen in the different components and dividing the sum with the total nitrogen analyses. A closer look at the contribution of the degradation compounds is given in Figures 11 and 12.

Figure 11 shows that overall the sum of nitrogen in different solvents and degradation compounds is the same as nitrogen given by total nitrogen analyses. However, for a few samples, the sum of nitrogen in the different compounds is slightly higher than 100%, caused by the use of different analytical instrumentation with different accuracies. Theoretically, the accuracy and uncertainties could be reduced using a deuterated internal standard for each compound. However, for the solvent blend studied here, it was not possible as deuterated internal standards were not available. From week 1 and throughout the campaign, the solvent compounds contributed between 96 and 98% to the nitrogen in the lean solvent samples, while the degradation compounds contributed between 0.4 and 1.3%, as seen in Figure 12. For the end sample (week 5), the deviation between the total *N* analysis and the sum of all *N* in the lean solvent from the determined compounds is 3%. This is not a significant difference when the uncertainties are also considered. Although the nitrogen balance for the lean solvent samples agrees, there still may be degradation compounds that are not identified.

**Figure 13:**
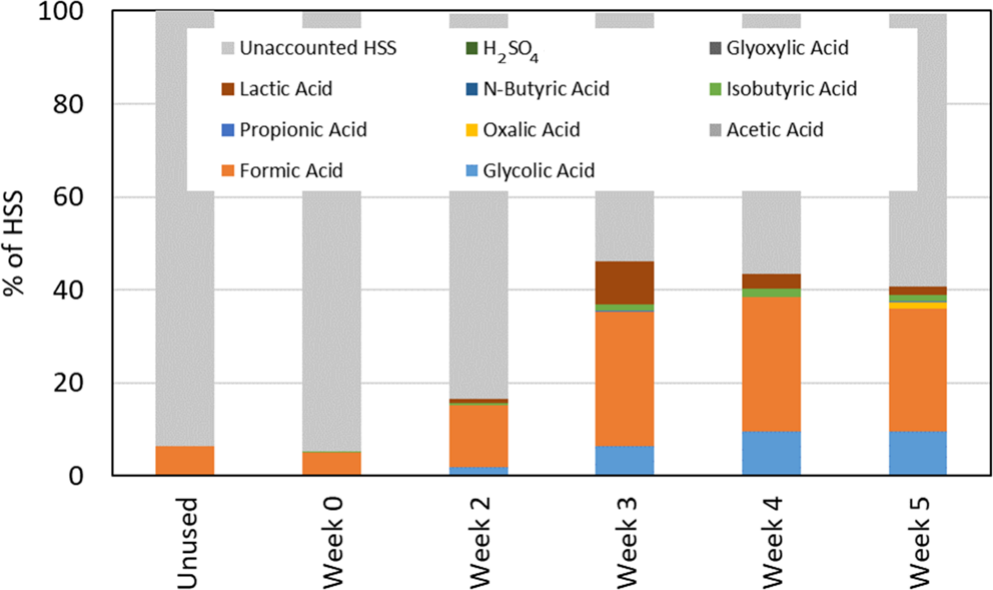
Acidic components (%) identified in the solvent samples that contribute to HSSs.

Total HSS and selected specific HSS compounds were also measured, and the results were close to LOQ (0.01 equiv/L), leading to relatively high uncertainty. However, an overview of the identified components and their contribution to the total HSS is given in Figure 13. The most significant contributions to HSS come from formic and glycolic acids. The acids identified are part of an analytical method used and are therefore not specific to this solvent system, and there might be solvent-specific acids that were not analyzed. Examples of solvent-specific acids could be 3-hydroxypropionic acid (503-66-2) and malonic acid (141-82-2) which are expected products from 3A1P.^33^

Nitrosamines were investigated using a total nitrosamine method and specific methods for different nitrosamines. The total nitrosamine method has higher uncertainty than the methods for the specific nitrosamines. A round Robin test for analyses of specific nitrosamine and total nitrosamine was previously reported by Fraboulet et al.,^34^ which showed considerable variation between different laboratories regarding their capabilities to analyze requested components and provide quantitative data. They concluded that the most reliable results were obtained for the specific nitrosamine in synthetic solutions and that the total nitrosamine method overestimated the results.

**Figure 14:**
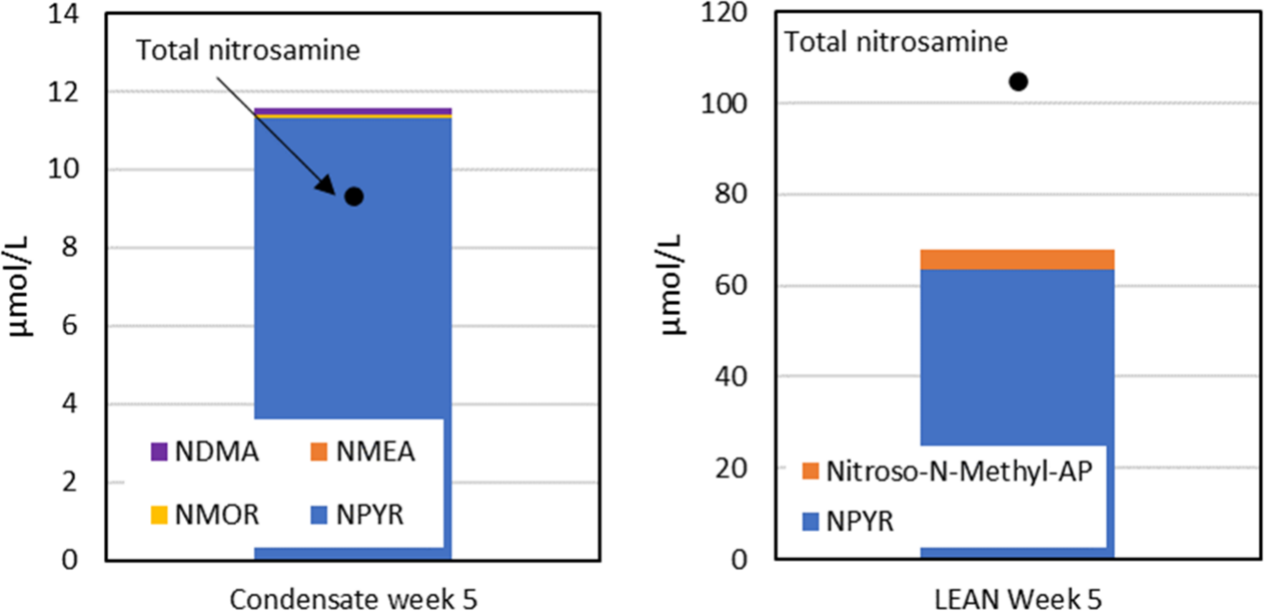
Concentration (μmol/L) of identified nitrosamines in lean week 5 on the left-hand side and condensate week 5 on the right-hand side.

NPYR was found to be the largest quantified, followed by nitroso-*N*-methyl-AP, as shown in Figure 14. For condensate samples, some of the typical nitrosamines such as NMOR, NMEA, and NDMA were also observed. However, the primary component is still NPYR. Higher uncertainty for the total nitrosamine methods is expected in solvent samples compared to condensate samples because of the high amine concentrations for the solvent samples. No other major nitrosamine is expected to be formed from the degradation compound quantified in this work. Thus, the gap between the total nitrosamine and the specific nitrosamine is believed to be due to analytical uncertainties. NOXZN, which potentially could have been formed from 3A1P, was not observed over the LOQ.

**Figure 15:**
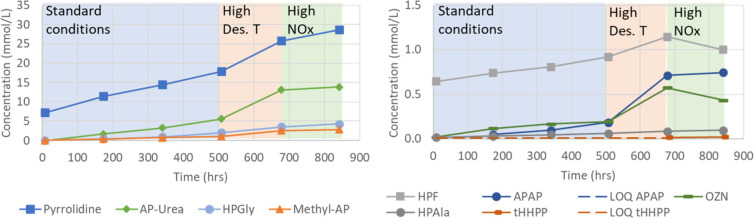
Concentration (mmol/L) of pyrrolidine, AP-urea, HPGly, Methyl-AP, HPF, APAP, OZN, HPAla, and tHHPP as a function of time (h) in the SDR campaign using three different conditions: (1) standard conditions [O_2_ (12%), stripper *T* (120 °C), and NOx (5 ppm)] shaded area in blue, (2) high stripper *T* [O_2_ (12%), stripper *T* (140 °C), and NOx (5 ppm)] shaded area in orange, and (3) higher NOx [O_2_ (12%), stripper *T* (120 °C), and NOx (50 ppm)] shaded area in green. LOQs for APAP and tHHPP are also given.

**Figure 16:**
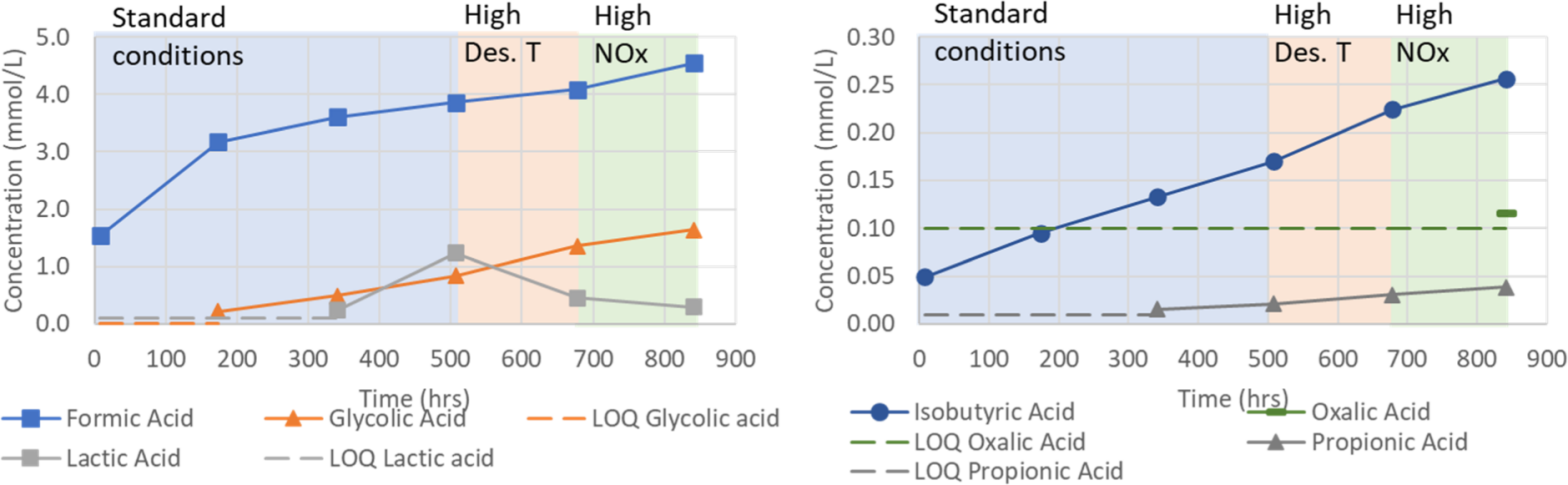
Concentration (mmol/L) of formic, glycolic, lactic, isobutyric, oxalic, and propionic acids as a function of time (h) in the SDR campaign using three different conditions: (1) standard conditions [O_2_ (12%), stripper *T* (120 °C), and NOx (5 ppm)] shaded area in blue, (2) high stripper *T* [O_2_ (12%), stripper *T* (140 °C), and NOx (5 ppm)] shaded area in orange, and (3) higher NOx [O_2_ (12%), stripper *T* (120 °C), and NOx (50 ppm)] shaded area in green. LOQs for APAP and tHHPP are also given.

The build-up of degradation compounds is a sum of the formation of initial products, the consumption of the formed degradation compound either through participation in other degradation reactions or decomposition, and the effect of potential equilibrium reactions as several of these degradation reactions may be equilibrium reactions. The degradation reactions occurring in the system cannot be isolated, and changing the experimental conditions, such as stripper temperature or NOx concentration, enforces a change in the chemical system. The build-up of the degradation compounds (mmol/L) in the solvent as a function of time (h) is shown in Figure 15 for degradation compounds with various functional groups (e.g., amide, amine, amino acids, and ring structures) and in Figure 16 for carboxylic acids.

A larger build-up rate at the higher desorber temperature is observed for many of the degradation compounds. The effect seems larger for components often categorized as thermal degradation compounds, such as AP-urea, methyl-AP, APAP, and OZN. The stepwise change in conditions influences the degradation ’compounds’ build-up or decomposition/consumption. Only methyl-AP and pyrrolidine seem to have similar build-up before and after the increased desorber temperature. For example, HPF and OZN, in the last week of the campaign, show larger decomposition/consumption rates than the initial formation rates under standard conditions. Most of the acids (formic, glycolic, propionic, isobutyric, HPAla, and HPGly) seem to have a smoother build-up (the initial week also, in this case, has a steeper build-up than the rest of the experiment). Some of the degradation compounds require time to accumulate in the solvent, for example, oxalic acid and tHHPP. This could be explained by oxalic acid being at the end of the oxidation route. It has also been observed to decompose at 135 °C.^35^ Higgins and co-workers investigated the thermal decomposition of oxalic acid (theoretically) to several products, such as formic acid and CO_2_.^36^ tHHPP is a six-membered ring that is expected to be relatively stable and not react with other products, and build-up over time is expected. However, several components on the pathway toward tHHPP also form other compounds, and varying the conditions may impact which reactions are more favorable.

**Figure 17:**
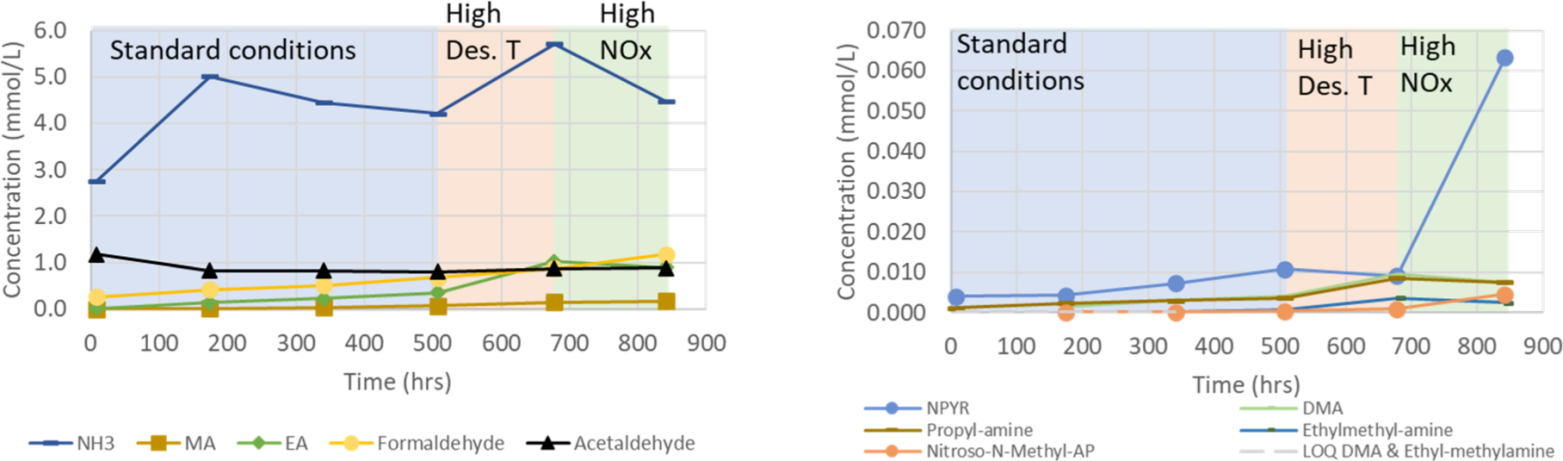
Concentration (mmol/L) of ammonia, MA, EA, formaldehyde, acetaldehyde, NPYR, DMA, propylamine, ethylmethylamine, and nitroso-*N*-methyl-AP as a function of time (h) in the SDR campaign using three different conditions: (1) standard conditions [O_2_ (12%), stripper *T* (120 °C), and NOx (5 ppm)] shaded area in blue, (2) high stripper *T* [O_2_ (12%), stripper *T* (140 °C), and NOx (5 ppm)] shaded area in orange, and (3) higher NOx [O_2_ (12%), stripper *T* (120 °C), and NOx (50 ppm)] shaded area in green. LOQs for DMA and ethylmethylamine are also given.

Concentrations of various volatile compounds that require monitoring from environmental aspects, such as ammonia, alkylamines, aldehydes, and nitrosamine, are given in Figure 17. As the SDR rig is a semiclosed rig, volatile components in the solvent will accumulate. Similar to MEA, ammonia, and formaldehyde are essential components for the studied blend. However, it is also clear that EA and acetaldehyde are important. These two components are typically low for MEA.^23,37^ The build-up of ammonia and alkylamine in this blend is lower than that for MEA under the same conditions.^23^ Compared to MEA, acetaldehyde and EA are found in the same order as formaldehyde, and EA is slightly more important than MA. Acetaldehyde could be explained by 1-(2HE)PRLD forming pyrrolidine and acetaldehyde by breaking the C–N bound. The blend consists of a tertiary and a primary amine; therefore, the hydrogen and the electron abstraction mechanism are expected to be important.^38−40^

The higher NOx condition was used to study the nitrosation of secondary amines to a nitrosamine. As discussed earlier, two nitrosamines were quantified in the solvent, one for each solvent component. It seems that a higher concentration of NPYR compared to *N*-nitroso-methyl-AP is explained by the higher concentration of pyrrolidine compared to methyl-AP. Pyrrolidine is the intermediate for the formation of NPYR, while methyl-AP is the intermediate for the formation of *N*-nitroso-methyl-AP. NPYR decreased at the higher stripper temperature (see Figure 17), and the decomposition of other cycled nitrosamines such as nitroso-piperazine was also observed at the stripper temperature.^41^ Interestingly, this is not the case for nitroso-*N*-methyl-AP.

### 3.4 Degradation Compound Pathways

A starting point for predicting degradation compounds for this particular blend was to adapt the typical degradation compounds from amines with similar molecular structures. Several degradation compounds formed from MEA result from MEA reacting with various degradation compounds. This means that in our solvent, the degradation compounds could react either with 3A1P or 1-(2HE)PRLD, creating degradation products that are a mix of both amines.

It is well accepted that oxidative degradation of amines in the process is initiated by a radical mechanism where oxygen can be an initiator or the O_2_ radical can participate directly. Exactly which of these happen is challenging to verify since the reactions are fast. In most cases, they only require another radical in close proximity for further propagation or termination of the reaction. Two types of mechanisms are generally suggested: the hydrogen abstraction mechanism and the electron abstraction mechanism. Studies suggest that primary amines with heteroatoms are more likely to go through the hydrogen abstraction mechanism, while tertiary amines undergo electron abstraction.^38−40^ However, both mechanisms lead to similar degradation compounds such as aldehydes, acids, ammonia, and alkylamines. Isotope marking could be one alternative for verifying how the amines split. From the molecular structure of 3A1P, it can be predicted that, for example, ammonia and propanol are formed by breaking the C–N bound (C–N scission step), while ethylamine and formaldehyde, methylamine, and acetaldehyde are formed by breaking the C–C bound (C–C scission step). The three aldehydes in the presence of oxygen will give formic, acetic, and propionic acid. Similarly, in 1-(2HE)PRLD solutions, glycolaldehyde, and pyrrolidine could be formed by breaking the C–N bound (C–N scission).

The compounds (ammonia, alkylamines, aldehydes, and acids) whose formation is initiated by radicals are often called primary degradation compounds. The secondary degradation compounds are the degradation compounds formed between the primary degradation compounds and the solvent amine(s) or other degradation compounds.

The characterization of degradation compounds for aqueous single amines or a blend of amines is often limited by the availability of advanced analytical instrumentation and commercially available standards for expected degradation compounds. Without commercially available standards, the presence in the solvent samples cannot be appropriately verified analytically, and the importance of these degradation compounds cannot be evaluated since no quantitative data can be given. For this work, the limitation was primarily related to expected degradation compounds from 1-(2HE)PRLD, where two (pyrrolidine and NPYR) of the four components expected were commercially available. The two other compounds suggested were expected to be formed by autooxidation. The autooxidation mechanism of *N*-alkyl pyrrolidine has been suggested by Beckwith et al.^42^ This has been adapted to 1-(2HE)PRLD and is given in Supporting Information in Scheme S1. For 3A1P, 10 compounds (methyl-AP, OZN, AP-urea, HPAla, HPF, tHHPP, APAP, nitroso-*N*-methyl-AP, NOXZN, and 5,6-dihydro-2-methyl-4*H*-1,3-oxazine—5638-63-1) were suggested based on the literature,^33^ with only one (5,6-dihydro-2-methyl-4*H*-1,3-oxazine) not being commercially available.

Additionally, two more components were suggested, 3-Mpy (a more general degradation compound) and HPGly (expected to be a product of the blend). In the initial characterization of the blend, other degradation compounds, which will be the results of both amines, were not included. This is partly because these compounds were not likely to be commercially available for verification since this is a new blend. Du,^43^ Namjoshi,^44^ and Du et al.^16^ have shown degradation pathways for blends that will also be relevant for this blend. Several smaller degradation compounds often observed in aqueous amine solvents, such as acids, acetone, aldehydes, ammonia, alkylamines, nitrosamines, and nitramine, were also included. In this work, the focus is the more solvent-specific degradation compounds. Despite this, a pathway for forming lactic, isobutyric, and propionic acid has been suggested and can be found in Supporting Information in Scheme S2. The pathways are adapted from a mechanism suggested by Gouedard^33^ for MEA as a solvent. 3-Mpy is a more general compound that can be formed from several amines as long as they form formaldehyde and acetaldehyde. Formaldehyde and acetaldehyde can then form acrolein, which can lead to 3-Mpy, as described in more detail elsewhere.^33^

**Scheme 1:**
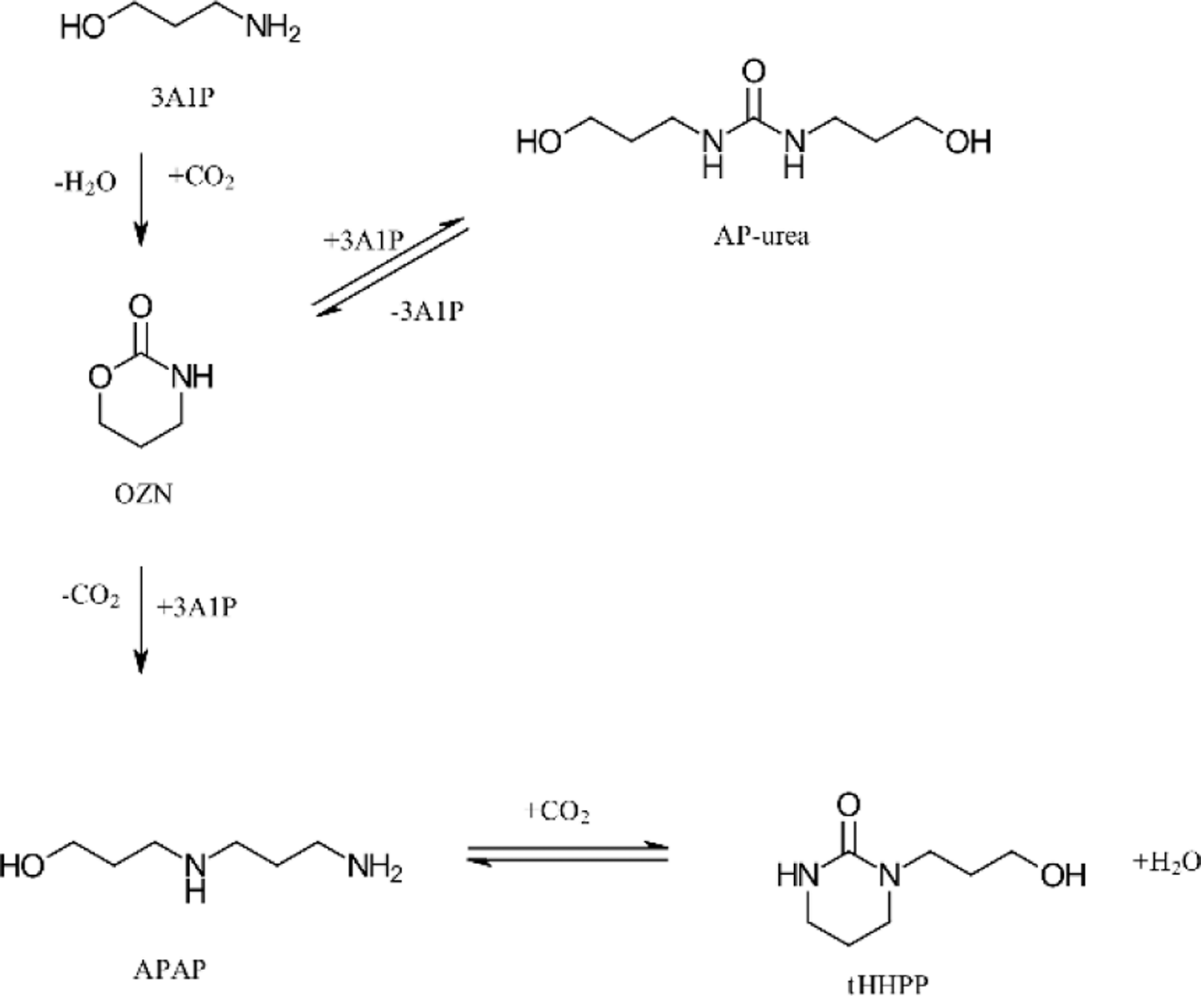
Suggested Pathway for the Formation of OZN, AP-Urea, APAP, and tHHPP Adapted from Literature^12,45,46^

For the secondary degradation, compounds such as OZN, AP-urea, APAP, and tHHPP following the pathway in Scheme 1 were adapted from Davis^12^ and Lepaumier et al.^45,46^ Several of these reactions consume CO_2_ and occur to a large extent at higher temperatures (e.g., stripper and reboiler). These are often called thermal degradation mechanisms or carbamate polymerization reactions.

**Scheme 2:**
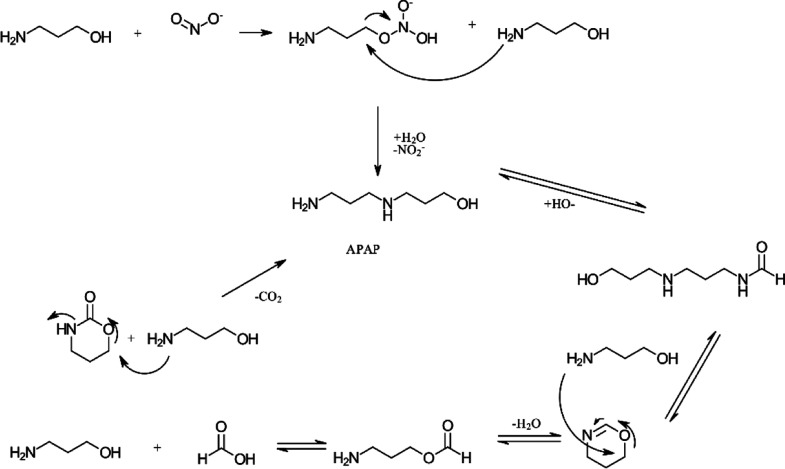
Suggested Pathways for the Formation of APAP Adapted from Literature^12,47,48^

Several formation routes have been suggested for forming the diamine from MEA (HEEDA—111-41-1), which is the analogue to APAP. Adapting these suggested reactions to 3A1P gives the following pathways (Scheme 2) for forming APAP.^45−48^

**Scheme 3:**
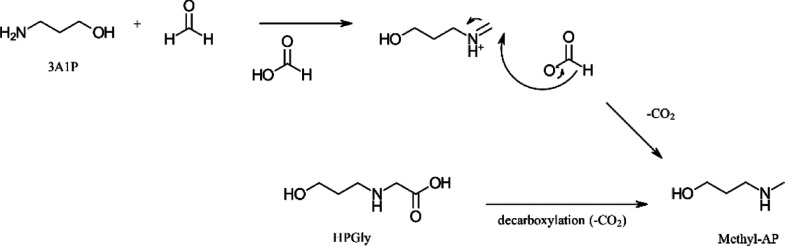
Suggested Pathways for the Formation of Methyl-AP Adapted from Literature^3,49−51^

For *N*-MeAP, several pathways could be suggested as well. For example, as given in Scheme 3 adapted from ref (49), *N*-MeAP formation happens through an Eschweiler–Clarke reaction^49^ or decarboxylation reaction from HPGly (decarboxylation of amino acids^33,50,51^). HPGly and methyl-AP were among the four most significant degradation compounds in the solvent samples from the SDR, see Figure 15.

**Scheme 4:**
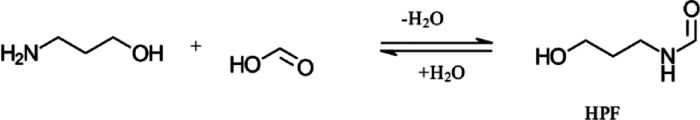
Suggested Pathway for the Formation of HPF Adapted from Lepaumier et al.^46^

For HPF, the suggested pathway is a reaction between 3A1P and formic acid, as shown in Scheme 4 adapted from Lepaumier et al.^46^

**Scheme 5:**
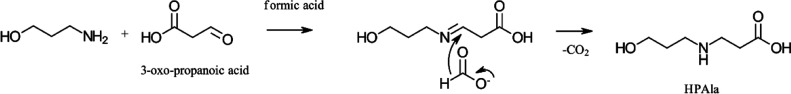
Suggested Pathway for the Formation of HPAla from 3-Oxo-propanoic Acid Adapted from Vevelstad et al.^24^

A pathway for HPAla is also suggested based on a pathway suggested for a similar compound formed from MEA.^24^ In this case, 3-oxo-propanoic acid is required in the reaction. A pathway suggested is shown in Scheme 5.

**Scheme 6:**
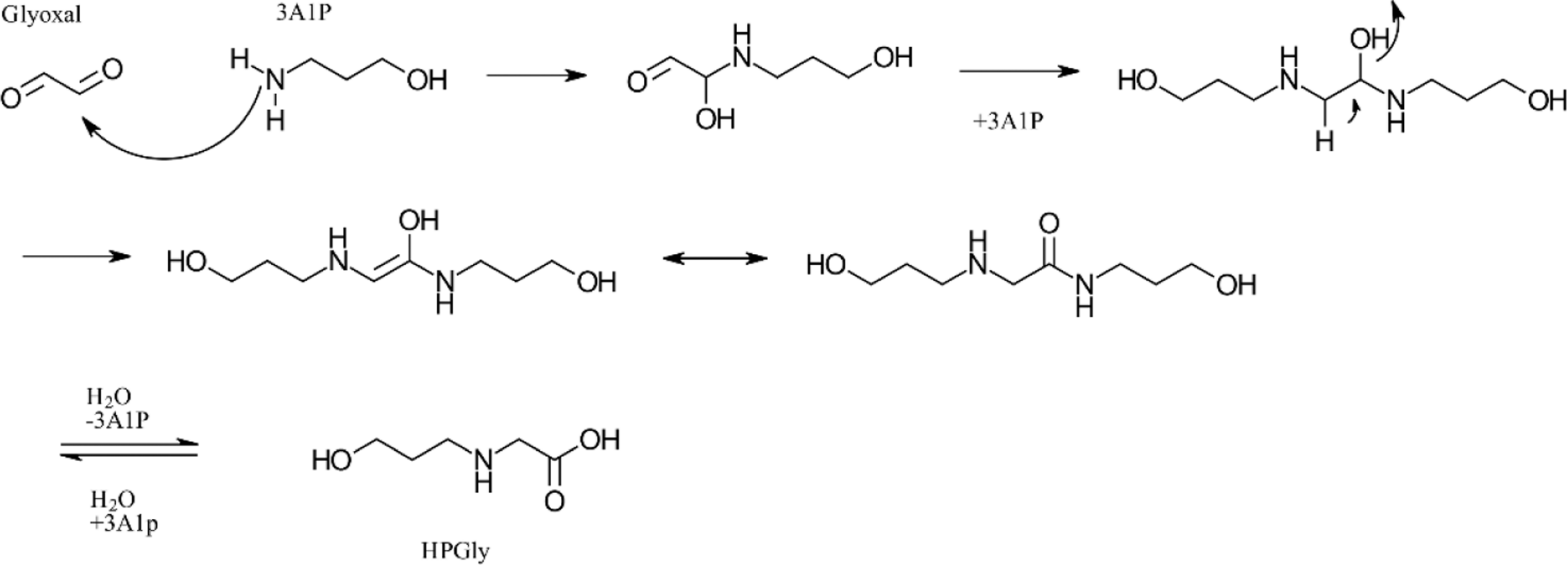
One of the Suggested Pathways for the Formation of HPGly Adapted from Gouedard^33^

HPGly could then either be formed from glyoxylic acid and 3A1P in a similar way as in Scheme 5 or from glyoxal and 3A1P as given in Scheme 6. This pathway is adapted from the pathway for MEA and glyoxal, as described by Gouedard.^33^

## 4 Summary/Conclusions

This work focuses on the chemical stability of 3A1P and 1-(2HE)PRLD blends and reports data on the solvent system under various conditions. Batch experiments were performed under conditions relevant to thermal degradation with CO_2_ and oxidative degradation. After that, the system was tested in a SDR where the solvent was cycled between the absorber and the desorber. Operating conditions were varied to investigate the robustness of the solvent to higher reboiler temperature and sensibility toward nitrosation.

The blend was found to be more stable than aqueous MEA and 3A1P in the thermal degradation experiment in the presence of CO_2_ but less stable than aqueous 1-(2HE)PRLD. For oxidative degradation experiments at 21% O_2_ and no metals added, the MEA, 3A1P, and the blend showed similar stability, while no or little loss was observed for 1-(2HE)PRLD. Higher oxygen concentration and metals present showed a high degradation of 1(2HE)PRLD. It was also shown that the chemical stability increased with the increase in promoter concentration under identical conditions. It is also clear that the presence of 3A1P reduces the stability of 1-(2HE)PRLD. The cyclic degradation experiment showed that the relative loss of the amines in the blend was comparable with MEA.

A thorough mapping of degradation compounds was conducted, resulting in quantifying 30 degradation compounds observed in solvent or condensate samples from the SDR. Twelve compounds were not observed over the LOQ; some of them could still be present in more degraded solvents, but the concentration of these components is expected to always be low compared to the other components quantified in this work. The main degradation components in the blend formed during oxidative degradation (96% O_2_ and metals present) were formic acid (formate), HPF, AP-urea, and OZN. Pyrrolidine and methyl-AP seemed to be impurities in the solvent with stable concentrations, not degradation compounds. In contrast, the major degradation components under cyclic conditions were ammonia, methyl-AP, AP-urea, pyrrolidine, formic acid (formate), and HPGly. Finally, formation pathways for eight solvent-specific degradation compounds (OZN, AP-urea, APAP, tHHPP, methyl-AP, HPF, HPAla, and HPGly) were suggested.

Overall, from a chemical stability perspective, this solvent blend is not significantly better than 30 wt % MEA, thus providing no benefits compared to MEA from the degradation point of view. Furthermore, in the studied blend, 1-(2HE)PRLD seems to degrade thermally to produce volatile pyrrolidine, which will form nitrosamine in the presence of NOx. Emission mitigation technologies to avoid, for example, pyrrolidine emissions, would be required.

## Supporting Information Available

Analytical data for all the performed degradation experiments; overview of the degradation compounds included in the analytical program for the SDR; and short description of analytical uncertainty and data treatment (PDF)

## Abbreviations

3A1P: 3-amino-1-propanol
1-(2HE)PRLD: 1-(2-hydroxyethyl)pyrrolidine
CESAR1: blend of piperazine (Pz) and 2-amino-2-methyl-1-propanol (AMP)
CCS: carbon capture and storage
GC-NCD: gas chromatography–nitrogen chemiluminescence detection
HSS: heat-stable salt
IC: ion chromatography
ICP-MS: inductively coupled plasma–mass spectrometry
LC–MS: liquid chromatography–mass spectrometry
LOQ: lower limit of quantification
MEA: monoethanolamine
MS: mass spectrometry
Pz: piperazine
SDR: solvent degradation rig
TIC: total inorganic carbon
TN: total nitrogen
TOC: total organic carbon
