# Supplementary material for: Chemical Stability and Characterization of Degradation Products of Blends of 1-(2-Hydroxyethyl)pyrrolidine and 3-Amino-1-propanol
Source: Ind Eng Chem Res. 2022 Dec 19;62(1):610–26. doi: 10.1021/acs.iecr.2c03068 (PMC9838088; doi:10.1021/acs.iecr.2c03068)
Supplement: Supplementary file 1 — ie2c03068_si_001.pdf [file ie2c03068_si_001.pdf]

Chemical stability and characterization of degradation products of blends of 1-(2-hydroxyethyl)pyrrolidine and 3-amino-1-propanol

Solrun Johanne Vevelstad[a], Andreas Grimstvedt[a], Maxime François[b], Hanna K. Knuutila[b*], Geir Haugen[a], Merete Wiig[a], Kai Vernstad[a]

[a]SINTEF Industry, 7465 Trondheim, Norway

[b]Department of Chemical Engineering, NTNU, NO-7491 , Norway

*Corresponding author: hanna.knuutila@ntnu.no

Supporting information

1.0 Analytical data for solvent samples from thermal degradation experiment

In the following section all data measured for the solvent samples from the thermal degradation experiment is given. This is the raw data (no correction has been made).

System 1-(2HE)PRLD – 40 wt% & 3A1P – 15 wt%

Table S 1. Operational/physical parameters (total amine by acid/base titration - amine eq/kg, CO_2 by total inorganic carbon /total organic carbon (TIC/TOC) analyzer - molCO_2/kg, density either by densitometer (Mettler-Toledo CM40) – g/mL

Sample ID	Date	Time	Week	Density<br>g/mL	Total Amine<br>amine eq/kg	CO2<br>mol CO_2/kg
Thermal, Week 0	06.06.2017	14:05	0	1.11	4.95	2.04
Thermal, Week 1	13.06.2017	07:50	1	NA	NA	NA
Thermal, Week 2	20.06.2017	07:58	2	NA	NA	NA
Thermal,Week 3	27.06.2017	07:40	3	NA	NA	NA
Thermal, Week 4	04.07.2017	08:30	4	NA	NA	NA
Thermal, Week 5	11.07.2017	07:40	5	1.10	4.62	1.83
Thermal, Week 5 #2	11.07.2017	07:40	5	1.10	4.61	1.82

Table S 2. Metals measured using ICP-MS – µg/L.

Sample ID	Cr		Fe		Ni	
	µg/L	RSD(%)	µg/L	RSD(%)	µg/L	RSD(%)
Thermal, Week 0	5	28	57	9	<5	-
Thermal, Week 1	35003	0.4	41991	0.5	23683	1.9
Thermal, Week 2	48644	0.1	33194	0.8	33012	0.6
Thermal,Week 3	61054	0.6	36073	0.3	41476	0.4
Thermal, Week 4	75687	1.1	37936	1.3	42908	1.3
THermal, Week 5	112449	0.7	41395	1.6	60444	0.9
Thermal, Week 5 #2	86120	0.4	35863	0.4	57911	1.1

Table S 3. Solvent amines (1-(2HE)PRLD & 3A1P) and degradation compound (pyrrolidine) measured using Liquid Chromatography -Mass Spectrometry (LC-MS) – g/L for solvent amines and mg/L for pyrrolidine.

Sample ID	2955-88-6<br>1-(2HE)PRLD<br>g/L	156-87-6<br>3A1P<br>g/L	123-75-1<br>Pyrrolidine<br>mg/L
Thermal, Week 0	402	149	117
Thermal, Week 1	393	134	2232
Thermal, Week 2	384	127	2749
Thermal, Week 3	378	120	3578
Thermal, Week 4	354	112	4333
Thermal, Week 5	347	108	4523
Thermal, Week 5 #2	347	107	4945

2.0 Analytical data for solvent samples from oxidative degradation experiments

In the following section all data measured for the solvent samples from the oxidative degradation experiments are given. This is the raw data (no correction has been made).

2.1 Setup 1 (21% O_2, α= 0.4 mol CO_2/mol amine, 55 °C)

System 1-(2HE)PRLD – 40 wt% & 3A1P – 15 wt%

Table S 4. Operational/physical parameters (total amine by acid/base titration - amine eq/kg, CO_2 by total inorganic carbon /total organic carbon (TIC/TOC) analyzer - molCO_2/kg, density by densitometer (Mettler-Toledo CM40) – g/mL, water using Karl Fisher titrator – wt%, organic nitrogen using Kjeldahl method – wt%. NA = not analyzed.

Sample ID	Date	Time	Days	Density<br>g/mL	Total Amine<br>amine eq/kg	CO2<br>mol CO2/kg	Water<br>wt%	Organic Nitrogen (Kjeldahl)<br>wt%
Setup1, day 0	06.06.17	14:10	0	1.11	4.93	2.02	40.5	6.90
Setup1, day 1	07.06.17	12:50	1	NA	NA	NA	41.2	NA
Setup1, day 3	09.06.17	13:25	3	NA	NA	NA	41.1	NA
Setup1, day 7	13.06.17	07:50	7	NA	NA	NA	41.9	NA
Setup1, day 10	16.06.17	08:45	10	NA	NA	NA	41.9	NA
Setup1, day 14	20.06.17	08:00	14	NA	NA	NA	42.0	NA
Setup1, day 17	23.06.17	08:15	17	NA	NA	NA	42.3	NA
Setup1, day 21	27.06.17	11:15	21	1.09	4.82	0.87	42.3	6.81
Gassbubbleflask, day 7	13.06.17	07:50	7	NA	NA	NA	NA	NA
Gassbubbleflask, day 14	20.06.17	08:00	14	NA	NA	NA	NA	NA
Gassbubbleflask, day 17	23.06.17	08:15	17	NA	NA	NA	NA	NA
Gassbubbleflask, day 21	27.06.17	11:15	21	NA	NA	NA	NA	NA

Table S 5. Solvent amines (1-(2HE)PRLD & 3A1P) degradation compounds (pyrrolidine, ammonia and NPYR) measured using LC-MS – solvent amine in g/L, pyrrolidine and ammonia in mg/L and NPYR in µg/L. NA = not analyzed.

Sample ID	2955-88-6<br>1-(2HE)PRLD<br>g/L	156-87-6<br>3A1P<br>g/L	123-75-1<br>Pyrrolidine<br>mg/L	7664-41-7<br>NH3<br>mg/L	930-55-2<br>NPYR<br>µg/L
Setup1, day 0	400	152	113	26	66
Setup1, day 1	392	151	171	NA	67
Setup1, day 3	388	151	244	NA	71
Setup1, day 7	372	148	346	21	74
Setup1, day 10	374	145	410	NA	93
Setup1, day 14	353	143	474	29	117
Setup1, day 17	347	142	533	NA	157
Setup1, day 21	333	139	566	28	205
Gassbubbleflask, day 7	0.055	0.041	0.53	376	0.30
Gassbubbleflask, day 14	0.17	0.11	2.5	585	0.80
Gassbubbleflask, day 17	0.11	0.073	2.5	503	0.10
Gassbubbleflask, day 21	0.086	0.056	2.1	352	1.0

2.2 Setup 2 (96% O_2, α= α= 0.4 mol CO_2/mol amine, 60 °C, 0.5 mM Fe)

2.2.1 System 1-(2HE)PRLD – 40 wt%

Table S 6. Solvent amines (1-(2HE)PRLD & 3A1P) measured using LC-MS – g/L (R1 = reactor 1 and R2 = reactor 2). System 1-2(HE)PRLD (40 wt%).

Sample id	2955-88-6<br>1-(2HE)PRLD<br>g/L	156-87-6<br>3A1P<br>g/L
Setup2, day 0	424	< 1
Setup2, day 6, R1	358	< 1
Setup2, day 14, R1	270	< 1
Setup2, day 21, R1	222	< 1
Setup2, day 6, R2	326	< 1
Setup2, day 14, R2	274	< 1
Setup2, day 21, R2	223	< 1

Table S 7. Degradation compounds measured using LC-MS – mg/L (R1 = reactor 1 and R2 = reactor 2). System 1-2(HE)PRLD (40 wt%).

Sample id	108-99-6<br>3-Mpy<br>mg/L	123-75-1<br>Pyrrolidine<br>mg/L
Setup2, day 0	< 10	9422
Setup2, day 6, R1	< 10	9168
Setup2, day 14, R1	< 10	8924
Setup2, day 21, R1	< 10	8649

Table S 8. Carboxylic acids measured using LC-MS – mg/L (R1 = reactor 1 and R2 = reactor 2). System 1-2(HE)PRLD (40 wt%).

Sample id	79-14-1<br>Glycolic Acid<br>mg/L	64-19-7<br>Acetic Acid<br>mg/L	79-09-4<br>Propionic Acid<br>mg/L	79-31-2<br>Isobutyric Acid<br>mg/L	107-92-6<br>N-Butyric Acid<br>mg/L	50-21-5<br>Lactic Acid<br>mg/L	298-12-4<br>Glyoxylic Acid<br>mg/L	64-18-6<br>Formic Acid<br>mg/L
Setup2, day 0	15	< 10	< 1	< 1	< 1	< 10	< 1	68
Setup2, day 6, R1	181	33	1.9	< 1	< 1	< 10	< 1	909
Setup2, day 14, R1	381	95	3.9	< 1	< 1	< 10	< 1	2283
Setup2, day 21, R1	488	153	5.6	< 1	< 1	< 10	< 1	3166

2.2.2 System 1-(2HE)PRLD – 40 wt% & 3A1P – 5 wt%

Table S 9. Solvent amines (1-(2HE)PRLD & 3A1P) measured using LC-MS – g/L (R1 = reactor 1 and R2 = reactor 2). System 1-2(HE)PRLD (40 wt%) & 3A1P (5 wt%).

Sample id	2955-88-6<br>1-(2HE)PRLD<br>g/L	156-87-6<br>3A1P<br>g/L
Setup2, day 0	389	51
Setup2, day 7, R1	280	39
Setup2, day 14, R1	243	39
Setup2, day 21, R1	169	31
Setup2, day 7, R2	312	45
Setup2, day 14, R2	231	39

Table S 10. Degradation compounds measured using LC-MS – mg/L (R1 = reactor 1 and R2 = reactor 2). System 1-2(HE)PRLD (40 wt%) & 3A1P (5 wt%).

Sample id	108-99-6<br>3-Mpy<br>mg/L	123-75-1<br>Pyrrolidine<br>mg/L	100747-20-4<br>HPGly<br>mg/L	42055-15-2<br>Methyl-AP<br>mg/L	5259-97-2<br>OZN<br>mg/L	71466-11-0<br>AP-Urea<br>mg/L	55937-35-4<br>HPala<br>mg/L	49807-74-1<br>HPF<br>mg/L	670227-88-0<br>tHHPP<br>mg/L	40226-15-1<br>APAP<br>mg/L
Setup2, day 0	< 10	7729	1.1	1.0	< 1	< 1	< 1	54	< 1	< 1
Setup2, day 7, R1	< 10	6642	32	< 1	18	16	9.5	447	< 1	< 1
Setup2, day 14, R1	< 10	7426	83	1.2	40	41	24	649	< 1	< 1
Setup2, day 21, R1	< 10	6634	117	< 1	52	57	36	665	< 1	< 1

Table S 11. Carboxylic acids measured using LC-MS – mg/L (R1 = reactor 1 and R2 = reactor 2). System 1-2(HE)PRLD (40 wt%) & 3A1P (5 wt%).

Sample id	79-14-1<br>Glycolic Acid<br>mg/L	64-19-7<br>Acetic Acid<br>mg/L	79-09-4<br>Propionic Acid<br>mg/L	79-31-2<br>Isobutyric Acid<br>mg/L	107-92-6<br>N-Butyric Acid<br>mg/L	50-21-5<br>Lactic Acid<br>mg/L	298-12-4<br>Glyoxylic Acid<br>mg/L	64-18-6<br>Formic Acid<br>mg/L
Setup2, day 0	< 1	< 10	< 1	< 1	< 1	< 10	< 1	43
Setup2, day 7, R1	24	20	1.3	< 1	< 1	< 10	< 1	533
Setup2, day 14, R1	55	41	2.4	< 1	< 1	< 10	< 1	1242
Setup2, day 21, R1	87	76	3.3	< 1	< 1	< 10	< 1	1790

2.2.3 System 1-(2HE)PRLD – 40 wt% & 3A1P – 15 wt%

Table S 12. Solvent amines (1-(2HE)PRLD & 3A1P) measured using LC-MS – g/L (R1 = reactor 1 and R2 = reactor 2). System 1-2(HE)PRLD (40 wt%) & 3A1P (15 wt%).

Sample id	2955-88-6<br>1-(2HE)PRLD<br>g/L	156-87-6<br>3A1P<br>g/L
Setup2, day 0	385	156
Setup2, day 6, R1	358	152
Setup2, day 14, R1	262	137
Setup2, day 21, R1	212	125
Setup2, day 6, R2	338	143
Setup2, day 14, R2	279	132
Setup2, day 21, R2	212	125

Table S 13. Degradation compounds measured using LC-MS – mg/L (R1 = reactor 1 and R2 = reactor 2). System 1-2(HE)PRLD (40 wt%) & 3A1P (15 wt%).

Sample id	108-99-6	123-75-1	100747-20-4	42055-15-2	5259-97-2	71466-11-0	55937-35-4	49807-74-1	670227-88-0	40226-15-1
	3-Mpy	Pyrrolidine	HPGly	Methyl-AP	OZN	AP-Urea	HPala	HPF	tHHPP	APAP
	mg/L	mg/L	mg/L	mg/L	mg/L	mg/L	mg/L	mg/L	mg/L	mg/L
Setup2, day 0	< 10	8787	1.8	8.9	< 1	< 1	< 1	60	< 1	< 1
Setup2, day 6, R1	< 10	9793	21	7.2	23	84	14	975	< 1	< 1
Setup2, day 14, R1	< 10	9975	65	7.1	56	212	32	1830	< 1	1.2
Setup2, day 21, R1	< 10	9960	110	8.0	79	337	61	2410	< 1	1.9

Table S 14. Carboxylic acids measured using LC-MS – mg/L (R1 = reactor 1 and R2 = reactor 2). System 1-2(HE)PRLD (40 wt%) & 3A1P (15 wt%).

Sample id	79-14-1	64-19-7	79-09-4	79-31-2	107-92-6	50-21-5	298-12-4	64-18-6
	Glycolic Acid	Acetic Acid	Propionic Acid	Isobutyric Acid	N-Butyric Acid	Lactic Acid	Glyoxylic Acid	Formic Acid
	mg/L	mg/L	mg/L	mg/L	mg/L	mg/L	mg/L	mg/L
Setup2, day 0	< 10	< 100	< 10	< 10	< 10	< 100	< 10	< 100
Setup2, day 6, R1	< 10	< 100	< 10	< 10	< 10	< 100	< 10	183
Setup2, day 14, R1	12	< 100	< 10	< 10	< 10	< 100	< 10	537
Setup2, day 21, R1	17	< 100	< 10	< 10	< 10	< 100	< 10	1008

2.2.4 System 1-(2HE)PRLD – 40 wt% & 3A1P – 20 wt%

Table S 15. Solvent amines (1-(2HE)PRLD & 3A1P) measured using LC-MS – g/L (R1 = reactor 1 and R2 = reactor 2). System 1-2(HE)PRLD (40 wt%) & 3A1P (20 wt%).

Sample id	2955-88-6	156-87-6
	1-(2HE)PRLD	3A1P
	g/L	g/L
Setup2, day 0	371	200
Setup2, day 7, R1	341	200
Setup2, day 14, R1	262	193
Setup2, day 21, R1	216	170
Setup2, day 7, R2	339	194
Setup2, day 14, R2	288	179
Setup2, day 21, R2	262	174

Table S 16. Degradation compounds measured using LC-MS – mg/L (R1 = reactor 1 and R2 = reactor 2). System 1-2(HE)PRLD (40 wt%) & 3A1P (20 wt%).

Sample id	108-99-6	123-75-1	100747-20-4	42055-15-2	5259-97-2	71466-11-0	55937-35-4	49807-74-1	670227-88-0	40226-15-1
	**3-Mpy**	**Pyrrolidine**	**HPGly**	**Methyl-AP**	**OZN**	**AP-Urea**	**HPala**	**HPF**	**tHHPP**	**APAP**
	**mg/L**	**mg/L**	**mg/L**	**mg/L**	**mg/L**	**mg/L**	**mg/L**	**mg/L**	**mg/L**	**mg/L**
Setup2, day 0	< 10	8258	< 1	11	< 1	< 1	< 1	64	< 1	< 1
Setup2, day 7, R1	< 10	8524	21	13	21	86	9.0	1064	< 1	1.3
Setup2, day 14, R1	< 10	9650	47	12	42	191	28	1762	< 1	2.3
Setup2, day 21, R1	< 10	9057	77	12	56	288	47	2235	< 1	3.3

Table S 17. Carboxylic acids measured using LC-MS – mg/L (R1 = reactor 1 and R2 = reactor 2). System 1-2(HE)PRLD (40 wt%) & 3A1P (20 wt%).

Sample id	79-14-1	64-19-7	79-09-4	79-31-2	107-92-6	50-21-5	298-12-4	64-18-6
	**Glycolic Acid**	**Acetic Acid**	**Propionic Acid**	**Isobutyric Acid**	**N-Butyric Acid**	**Lactic Acid**	**Glyoxylic Acid**	**Formic Acid**
	**mg/L**	**mg/L**	**mg/L**	**mg/L**	**mg/L**	**mg/L**	**mg/L**	**mg/L**
Setup2, day 0	< 10	< 100	< 10	< 10	< 10	< 100	< 10	< 100
Setup2, day 7, R1	< 10	< 100	< 10	< 10	< 10	< 100	< 10	153
Setup2, day 14, R1	< 10	< 100	< 10	< 10	< 10	< 100	< 10	356
Setup2, day 21, R1	11	< 100	< 10	< 10	< 10	< 100	< 10	576

3.0 Analytical data for solvent samples from the SDR rig (raw data)

In the following section all data measured for the condensate sample and all the solvent samples from the SDR rig. This is the raw data (no correction has been made).

Table S 18. Operational/physical parameters (total amine by acid/base titration - amine eq/kg, CO_2 by total inorganic carbon /total organic carbon (TIC/TOC) analyzer - molCO_2/kg, density either by densitometer (Mettler-Toledo CM40) or gravimetrically (italic numbers) – g/mL, water using Karl Fisher titrator – wt%, total HSS using a wet chemistry method based on ion exchange and followed with by titration with NaOH – eq/kg and total nitrogen was determined by oxidative catalytic combustion and chemiluminescence detection (Shimadzu TOC_L CHP TNM-L) – mg N/kg. Grey shading - Unused/virgin solvent and lean week 0 (introduced to the rig, but no CO_2); yellow shading - standard conditions (desorber T 120 °C, NOx 5 ppm); blue shading – high desorber T (desorber T 140 °C); green shading – high NOx (NOx 50 ppm). NA = not analyzed.

Sample id	Date	Time	hrs	Total Amine [amine eq/kg]	CO_2 (TIC) [mol CO_2/kg]	ρ [g/mL]	H_2O [wt%]	Total HSS [eq/kg]	Total N mg N/kg
Unused (virgin solvent)	04.01.2021	12:00	0	5.54	<0.025	*1.01*	45.2	0.03	77 502
Lean Week 0 unloaded (introduced to rig, but no CO2)	04.01.2021	12:00	0	5.27	<0.025	1.02	47.8	NA	74 950
LEAN Week 0	04.01.2021	19:40	8	5.15	0.64	*1.04*	46.0	0.03	74 153
LEAN Week 1	11.01.2021	17:40	174	5.10	0.70	*1.04*	46.6	NA	73 570
LEAN Week 2	18.01.2021	16:50	341	5.01	0.71	*1.04*	46.4	0.03	74 122
LEAN Week 3	25.01.2021	15:10	507	5.05	0.69	*1.04*	46.6	0.01	73 469
LEAN Week 4	01.02.2021	17:55	678	4.97	0.66	*1.04*	47.5	0.01	71 700
LEAN Week 5	08.02.2021	13:12	841	4.97	0.71	1.05	47.5	0.02	72 271
Condensate week 5	08.02.2021	11:18	839	NA	0.15	*1.00*	100	NA	2 373

Table S 19. Metals measured using ICP-MS – mg/L. Grey shading - Unused/virgin solvent and lean week 0 (introduced to the rig, but no CO_2); yellow shading - standard conditions (desorber T 120 °C, NOx 5 ppm); blue shading – high desorber T (desorber T 140 °C); green shading – high NOx (NOx 50 ppm).

Sample id	Fe [mg/L]	Ni [mg/L]	Cr [mg/L]	Cu [mg/L]	S [mg/L]	Zn [mg/L]	Ba [mg/L]	V [mg/L]	Na [mg/L]	Al [mg/L]
Unused (virgin solvent)	0.0083	0.0030	0.0026	0.034	< 0.1	0.013	< 0.01	< 0.0001	3.7	0.039
Lean Week 0 unloaded (introduced to rig. but no CO2)	0.016	0.0031	0.0026	0.10	< 0.1	0.021	< 0.01	< 0.0001	3.4	0.061
LEAN Week 0	0.28	0.36	0.044	0.28	0.14	0.060	0.019	0.00013	3.5	0.154
LEAN Week 1	0.40	0.15	0.13	0.27	0.73	0.611	0.024	0.00050	2.7	0.090
LEAN Week 2	0.36	0.18	0.15	0.23	1.1	0.869	0.030	0.00068	3.1	0.047
LEAN Week 3	0.36	0.19	0.17	0.17	1.3	1.05	0.026	0.00076	4.9	0.020
LEAN Week 4	0.94	0.22	0.21	0.10	1.6	1.32	0.025	0.0010	6.7	0.027
LEAN Week 5	0.60	0.23	0.22	0.088	1.8	1.44	0.018	0.0011	6.3	< 0.01
Condensate week 5	0.0012	0.0032	0.0011	< 0.01	< 0.1	< 0.01	0.031	< 0.0001	3.6	< 0.01

Table S 20. Solvent amines (1-(2HE)PRLD & 3A1P) measured using Liquid Chromatography -Mass Spectrometry (LC-MS) – g/kg. Grey shading - Unused/virgin solvent and lean week 0 (introduced to the rig, but no CO_2); yellow shading - standard conditions (desorber T 120 °C, NOx 5 ppm); blue shading – high desorber T (desorber T 140 °C); green shading – high NOx (NOx 50 ppm).

Sample id	2955-88-6<br>1-(2HE)PRLD<br>g/kg	156-87-6<br>3A1P<br>g/kg
Unused (virgin solvent)	412	155
Lean Week 0 unloaded (introduced to rig, but no CO2)	398	144
LEAN Week 0	390	140
LEAN Week 1	381	137
LEAN Week 2	376	136
LEAN Week 3	377	138
LEAN Week 4	374	134
LEAN Week 5	370	131
Condensate week 5	13	0.047

Table S 21. Overview of the degradation compounds which were included in the analytical program for the SDR experiment together with information if they were present and where they were present (solvent, condensate or both).

Functional groups	Abbreviation	Quantified (S)	Quantified (C)	Lower than LOQ
	Oxalic Acid	x		
	Formic Acid	x		
	Glycolic Acid	x		
	Acetic Acid			x
	Propionic Acid	x		
	Isobutyric Acid	x		
	N-Butyric Acid			x
	Lactic Acid	x		
Acids	Glyoxylic Acid			x
Ammonia	NH3	x	x	
	MA	x	x	
	EA	x	x	
	Propyl-amine	x	x	
	DMA	x	x	
	Ethylmethyl-amine	x	x	
	DiEA		x	
Alkylamine	Dipropyl-amine			x
	NDELA			x
	NDMA		x	
	NDEA			x
	NPIP			x
	NMEA		x	
	NPYR	x	x	
	NMOR		x	
	NDPA			x
	NDBA			x
	Nitroso-N-Methyl-AP	x		
Nitrosamine	tetrahydro-3-nitroso-2H-1,3-Oxazine			x
Nitramine	DMA-NO2			x
	Formaldehyde	x	x	
Aldehyde	Acetaldehyde	x	x	
Ketone	Acetone			x
	Methyl-AP	x	x	
	APAP	x		
Amine (secondary)	Pyrrolidine	x	x	
Amide	HPF	x	x	
Urea	AP-Urea	x		
	HPAla	x	x	
Aminoacids	HPGly	x	[a]	
	OZN	x		
	3-Mpy		x	
Various rings structures	tHHPP	x		
Sum	42	25	18	12

a: Not enough sample left

Table S 22. Ammonia and alkylamine measured using LC-MS – mg/kg. Grey shading - Unused/virgin solvent and lean week 0 (introduced to the rig, but no CO_2); yellow shading - standard conditions (desorber T 120 °C, NOx 5 ppm); blue shading – high desorber T (desorber T 140 °C); green shading – high NOx (NOx 50 ppm).

**Sample id**	**7664-41-7**<br>**NH3**<br>**mg/kg**	**74-89-5**<br>**MA**<br>**mg/kg**	**75-04-7**<br>**EA**<br>**mg/kg**	**107-10-8**<br>**Propyl-amine**<br>**mg/kg**	**124-40-3**<br>**DMA**<br>**mg/kg**	**624-78-2**<br>**Ethylmethyl-amine**<br>**mg/kg**	**109-89-7**<br>**DiEA**<br>**mg/kg**	**142-84-7**<br>**Dipropyl-amine**<br>**mg/kg**
Unused (virgin solvent)	20.3	< 0.010	0.19	0.07	< 0.010	< 0.010	< 0.10	< 0.10
Lean Week 0 unloaded (introduced to rig, but no CO2)	21	< 0.010	0.22	0.068	< 0.010	< 0.010	< 0.10	< 0.10
LEAN Week 0	45	0.046	0.38	0.063	< 0.010	< 0.010	< 0.10	< 0.10
LEAN Week 1	82	0.48	5.8	0.13	0.067	< 0.010	< 0.10	< 0.10
LEAN Week 2	73	1.1	10	0.17	0.13	0.017	< 0.10	< 0.10
LEAN Week 3	69	2.1	15	0.20	0.17	0.032	< 0.10	< 0.10
LEAN Week 4	93	4.5	44	0.49	0.41	0.20	< 0.10	< 0.10
LEAN Week 5	73	5.0	39	0.43	0.33	0.14	< 0.10	< 0.10
Condensate week 5	1062	3.4	90	1.0	1.5	0.99	1.1	< 0.001

Table S 23. Formaldehyde, acetaldehyde and acetone measured using LC-MS – mg/kg. Grey shading - Unused/virgin solvent and lean week 0 (introduced to the rig, but no CO_2); yellow shading - standard conditions (desorber T 120 °C, NOx 5 ppm); blue shading – high desorber T (desorber T 140 °C); green shading – high NOx (NOx 50 ppm).

**Sample id**	**50-00-0**<br>**Formaldehyde**<br>**mg/kg**	**75-07-0**<br>**Acetaldehyde**<br>**mg/kg**	**67-64-1**<br>**Acetone**<br>**mg/kg**
Unused (virgin solvent)	23.7	29.2	< 5
Lean Week 0 unloaded (introduced to rig, but no CO2)	23	29	< 5
LEAN Week 0	7.6	50	< 5
LEAN Week 1	12	35	< 5
LEAN Week 2	15	35	< 5
LEAN Week 3	20	34	< 5
LEAN Week 4	25	37	< 5
LEAN Week 5	34	37	< 5
Condensate week 5	0.037	0.20	< 0.25

Table S 24. Total nitrosamine measured using Gas Chromatography – Nitrogen Chemiluence Detector (GC-NCD) – µmol/kg and nitramine (DMA-NO2) measured using LC-MS – mg/kg. Grey shading - Unused/virgin solvent and lean week 0 (introduced to the rig, but no CO_2); yellow shading - standard conditions (desorber T 120 °C, NOx 5 ppm); blue shading – high desorber T (desorber T 140 °C); green shading – high NOx (NOx 50 ppm). NA = not analyzed.

Sample id	Tot nitrosamin<br>µmol/kg	4164-28-7<br>DMA-NO2<br>mg/kg
Unused (virgin solvent)	< 40	< 1
Lean Week 0 unloaded (introduced to rig, but no CO2)	NA	< 1
LEAN Week 0	< 40	< 1
LEAN Week 1	< 40	< 1
LEAN Week 2	NA	< 1
LEAN Week 3	< 40	< 1
LEAN Week 4	< 40	< 1
LEAN Week 5	100	< 1
Condensate week 5	9.3	< 0.002

Table S 25. Nitrosamine measured using LC-MS – µg/kg. Grey shading - Unused/virgin solvent and lean week 0 (introduced to the rig, but no CO_2); yellow shading - standard conditions (desorber T 120 °C, NOx 5 ppm); blue shading – high desorber T (desorber T 140 °C); green shading – high NOx (NOx 50 ppm).

Sample id	1116-54-7<br>NDELA<br>µg/kg	62-75-9<br>NDMA<br>µg/kg	55-18-5<br>NDEA<br>µg/kg	100-75-4<br>NPIP<br>µg/kg	10595-95-6<br>NMEA<br>µg/kg	930-55-2<br>NPYR<br>µg/kg	59-89-2<br>NMOR<br>µg/kg	621-64-7<br>NDPA<br>µg/kg	924-16-3<br>NDBA<br>µg/kg	70415-59-7<br>Nitroso-N-Methyl-AP<br>µg/kg	35627-29-3<br>NOXZN<br>µg/kg
Unused (virgin solvent)	< 5	< 5	< 5	< 5	< 5	346	< 5	< 5	< 5	< 5	<10
Lean Week 0 unloaded (introduced to rig, but no CO2)	< 5	< 5	< 5	< 5	< 5	335	< 5	< 5	< 5	< 5	<10
LEAN Week 0	< 5	< 5	< 5	< 5	< 5	383	< 5	< 5	< 5	< 5	<10
LEAN Week 1	< 5	< 5	< 5	< 5	< 5	415	< 5	< 5	< 5	6.1	<10
LEAN Week 2	< 5	< 5	< 5	< 5	< 5	702	< 5	< 5	< 5	18.6	<10
LEAN Week 3	< 5	< 5	< 5	< 5	< 5	1 032	< 5	< 5	< 5	42.2	<10
LEAN Week 4	< 5	< 5	< 5	< 5	< 5	868	< 5	< 5	< 5	103	<10
LEAN Week 5	< 5	< 5	< 5	< 5	< 5	6 060	< 5	< 5	< 5	520	<10
Condensate week 5	< 0.5	12	< 0.5	< 0.5	1.6	1 133	8.1	< 0.5	< 0.5	< 0.5	<1

Table S 26. Various solvent specific degradation compounds measured using LC-MS – mg/kg. Grey shading - Unused/virgin solvent and lean week 0 (introduced to the rig, but no CO_2); yellow shading - standard conditions (desorber T 120 °C, NOx 5 ppm); blue shading – high desorber T (desorber T 140 °C); green shading – high NOx (NOx 50 ppm). NA = not analyzed.

Sample id	108-99-6<br>3-Mpy<br>mg/kg	123-75-1<br>Pyrrolidine<br>mg/kg	100747-20-4<br>HPGly<br>mg/kg	42055-15-2<br>Methyl-AP<br>mg/kg	5259-97-2<br>OZN<br>mg/kg	71466-11-0<br>AP-Urea<br>mg/kg	55937-35-4<br>HPAla<br>mg/kg	49807-74-1<br>HPF<br>mg/kg	670227-88-0<br>tHHPP<br>mg/kg	40226-15-1<br>APAP<br>mg/kg
Unused (virgin solvent)	< 1	442	< 5	5.2	< 1	< 0,1	1.4	8.2	< 1	< 1
Lean Week 0 unloaded (introduced to rig, but no CO2)	< 1	440	< 5	5.2	< 1	< 0.1	1.7	7.4	< 1	< 1
LEAN Week 0	< 1	491	8.3	6.9	1.7	2.1	2.2	64	< 1	< 1
LEAN Week 1	< 1	783	53	32	11	282	4.5	73	< 1	6.1
LEAN Week 2	< 1	989	117	64	16	552	6.4	80	< 1	12
LEAN Week 3	< 1	1 216	256	92	19	938	8.5	91	< 1	23
LEAN Week 4	< 1	1 756	447	218	56	2 204	12	113	2.8	90
LEAN Week 5	< 1	1 945	544	243	42	2 330	13	98	3.2	94
Condensate week 5	0.55	29	NA	0.62	< 0.002	< 0.0002	0.019	0.10	< 0.01	< 0.01

Table S 27. Carboxylic acids measured using LC-MS – mg/kg. Grey shading - Unused/virgin solvent and lean week 0 (introduced to the rig, but no CO_2); yellow shading - standard conditions (desorber T 120 °C, NOx 5 ppm); blue shading – high desorber T (desorber T 140 °C); green shading – high NOx (NOx 50 ppm). NA = not analyzed.

Sample id	79-14-1<br>Glycolic Acid<br>mg/kg	64-19-7<br>Acetic Acid<br>mg/kg	79-09-4<br>Propionic Acid<br>mg/kg	79-31-2<br>Isobutyric Acid<br>mg/kg	107-92-6<br>N-Butyric Acid<br>mg/kg	50-21-5<br>Lactic Acid<br>mg/kg	298-12-4<br>Glyoxylic Acid<br>mg/kg
Unused (virgin solvent)	< 1	< 10	< 1	< 1	< 1	< 10	< 1
Lean Week 0 unloaded (introduced to rig, but no CO2)	< 1	< 10	< 1	4.3	< 1	< 10	< 1
LEAN Week 0	< 1	< 10	< 1	4.2	< 1	< 10	< 1
LEAN Week 1	16	< 10	< 1	8.0	< 1	< 10	< 1
LEAN Week 2	36	< 10	1.1	11	< 1	21	< 1
LEAN Week 3	61	< 10	1.5	14	< 1	107	< 1
LEAN Week 4	99	< 10	2.2	19	< 1	39	< 1
LEAN Week 5	119	< 10	2.7	22	< 1	26	< 1
Condensate week 5	NA	NA	NA	NA	NA	NA	NA

Table S 28. Formate and oxalate measured using Ion Chromatography (IC) – mg/kg. Grey shading - Unused/virgin solvent and lean week 0 (introduced to the rig, but no CO_2); yellow shading - standard conditions (desorber T 120 °C, NOx 5 ppm); blue shading – high desorber T (desorber T 140 °C); green shading – high NOx (NOx 50 ppm). NA = not analyzed.

Sample id	71-47-6 Formate mg/kg	338-70-5 Oxalate mg/kg
Unused (virgin solvent)	78	<10
Lean Week 0 unloaded (introduced to rig, but no CO2)	84	<10
LEAN Week 0	68	<10
LEAN Week 1	140	<10
LEAN Week 2	160	<10
LEAN Week 3	170	<10
LEAN Week 4	180	<10
LEAN Week 5	200	10
Condensate week 5	NA	NA

3.1 Data treatment and experimental and analytical uncertainty

By using the law of error propagation, the uncertainty in the corrected concentration could be derived based on the total differential of Eq [1] in the paper, which yield:

$$\Delta C_i^{Corr} = \sqrt{\left(\frac{\partial C_i^{Corr}}{\partial Corr_{factor}}\right)^2 \cdot \Delta Corr_{factor}{}^2 + \left(\frac{\partial C_i^{Corr}}{\partial C_i^w}\right)^2 \cdot \Delta C_i^{w\,2}} \qquad [4]$$

$$\Delta C_i^{Corr} = \sqrt{C_i^{w\,2}\left\{\left[\frac{\Delta(\Delta X_{H2O})}{(1-\Delta X_{H2O}-\Delta X_{CO2})^2}\right]^2 + \left[\frac{\Delta(\Delta X_{CO2})}{(1-\Delta X_{H2O}-\Delta X_{CO2})^2}\right]^2\right\} + \frac{\Delta C_i^{w\,2}}{(1-\Delta X_{H2O}-\Delta X_{CO2})^2}} \qquad [5]$$

This equation could be rearranged somewhat to yield the relative uncertainty

$$\left(\frac{\Delta C_i^{Corr}}{C_i^{Corr}}\right) = \sqrt{[\Delta(\Delta X_{H2O})]^2 + [\Delta(\Delta X_{CO2})]^2 + \left(\frac{\Delta C_i^w}{C_i^w}\right)^2} \qquad [6]$$

This can be further simplified by introducing the uncertainty in the water difference between the actual and reference (R) sample and assuming the same uncertainty (σ_X) for both $X_{i,H2O}$ and $X_{R,H20}$. For reference sample usually the first sample could be chosen (i.e. correction factor of this sample will then be 1, and correction factor of the other is dependent of the difference in water and CO_2 content to this reference). By the assumption above the contribution from water becomes :

$$[\Delta(\Delta X_{H2O})]^2 = \sigma^2_{X_{i,H2O}} + \sigma^2_{X_{R,H2O}} \approx 2\sigma^2_{X\ H2O} \qquad [7]$$

Similar can be done for the uncertainty in CO_2, by introducing this into Eq. [6] an expression for the relative uncertainty of the corrected concentration as a combination of the relative uncertainty in concentrations of CO_2, H_2O and the compound of interest (i) is obtained.

$$\left(\frac{\Delta C_i^{Corr}}{C_i^{Corr}}\right) = \sqrt{2\left(\sigma^2_{X_{H2O}} + \sigma^2_{X_{CO2}}\right) + \left(\frac{\Delta C_i^{w}}{C_i^{w}}\right)^2} = \sqrt{2\left[\left(\frac{\Delta C^{w}_{H_2O}}{C^{w}_{H_2O}}\right)^2 + \left(\frac{\Delta C^{w}_{CO_2}}{C^{w}_{CO_2}}\right)^2\right] + \left(\frac{\Delta C_i^{w}}{C_i^{w}}\right)^2} \qquad [8]$$

If then the relative uncertainty of water is 1.0%, CO_2 2.5% and 5.0% for the compound of interest (on weight basis), the relative uncertainty of the corrected concentration will be 6.3% as calculated in Eq. [9].

$$\left(\frac{\Delta C_i^{Corr}}{C_i^{Corr}}\right) = \sqrt{2[(0.010)^2 + (0.025)^2] + (0.050)^2} = 0.063 \qquad [9]$$

4.0 Degradation pathways

Scheme S 1. Autooxidation of 1-(2HE)PRLD, adapted to the autooxidation mechanism for N-alkylpyrrolidine suggested by Beckwith et al. [1]

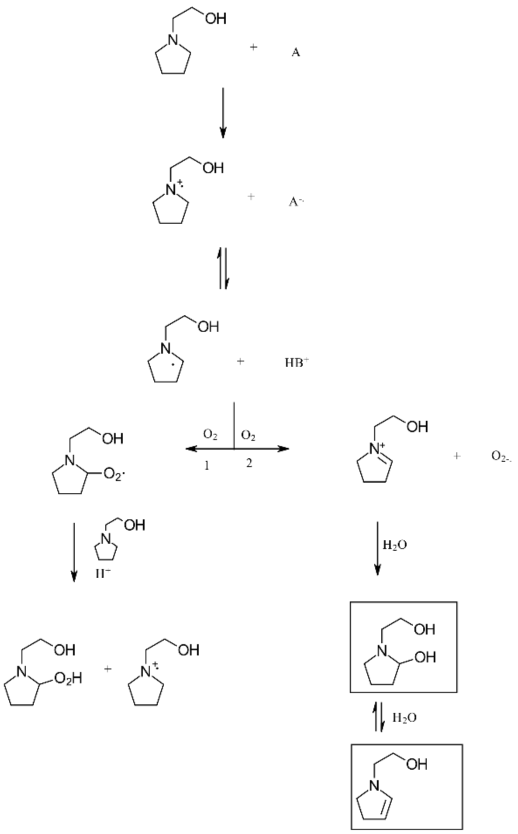

For this blend, there were some surprises for the acids. Lactic acid has been suggested as degradation compound from MEA but has rarely been observed. In this work, both lactic and iso-butyric acid were

quantified. A pathway for formation of these and propionic acid is given below. This mechanism for formation of lactic and propionic acid from acetaldehyde and formaldehyde were suggested by Gouedard[2]. The suggested mechanism for iso-butyric is adapted from this mechanism, where the formaldehyde and 3-hydroxypropanal is the start material.

Scheme S 2. Suggested pathway for lactic acid, propionic acid and iso-butyric acid

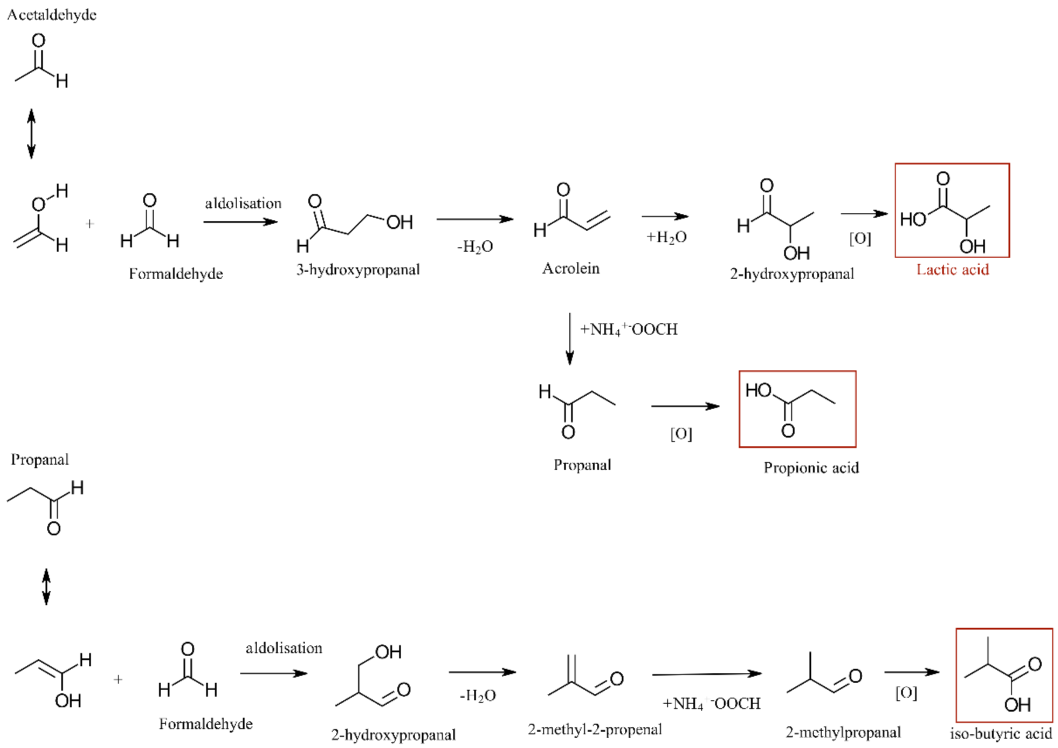

Acknowledgement

This work was performed within the HiPerCap, REALISE and LAUNCH project. The project has received funding from the European Union's Horizon 2020 research and innovation programme under grant agreement No 884266 (REALISE project), the European Unio Seventh Framework Programme (FP7/2007-2013) under grant agreement No 608555 (HiPerCap project). The industrial partners who also financially support the project are gratefully acknowledged.

The LAUNCH project (Project No. 299662) is co-funded by the ERA-NET Accelerating CCS Technologies iniative, which supports the delivery of safe and cost-effective carbon capture, utilisation and storage. The government of each participating country have contributed funding through the ACT2 iniative.

References

(1) Beckwith, A.; Eichinger, P.; Mooney, B.; Prager, R. Amine autoxidation in aqueous solution. *Australian Journal of Chemistry* **1983**, *36* (4), 719-739. DOI: https://doi.org/10.1071/CH9830719.

(2) Gouedard, C. Novel degradation products of ethanolamine (MEA) in CO_2 capture conditions: identification, mechanisms proposal and transportation to other amines. Universite Pierre et Marie Curie, 2014.
